# ZntR is a critical regulator for zinc homeostasis and involved in pathogenicity in *Riemerella anatipestifer*

**DOI:** 10.1128/spectrum.03178-24

**Published:** 2025-03-04

**Authors:** Hongmeng Ma, Mengying Wang, Yizhou Yao, Shutong Zhang, Mingshu Wang, Dekang Zhu, Renyong Jia, Shun Chen, Xinxin Zhao, Qiao Yang, Ying Wu, Shaqiu Zhang, Juan Huang, Bin Tian, Xumin Ou, Di Sun, Yu He, Zhen Wu, Ling Zhang, Yanling Yu, Anchun Cheng, Mafeng Liu

**Affiliations:** 1 Engineering Research Center of Southwest Animal Disease Prevention and Control Technology, Ministry of Education of the People’s Republic of China, Chengdu, China; 2 Key Laboratory of Animal Disease and Human Health of Sichuan Province, Chengdu, China; 3 International Joint Research Center for Animal Disease Prevention and Control of Sichuan Province, Chengdu, China; 4 Research Center of Avian Disease, College of Veterinary Medicine, Sichuan Agricultural University, Chengdu, China; Griffith University-Gold Coast Campus, Gold Coast, Australia

**Keywords:** zinc homeostasis, *R. anatipestifer*, regulator, pathogenicity

## Abstract

Zinc (Zn^2+^) is essential for all bacteria, but excessive Zn^2+^ levels are toxic. Bacteria maintain zinc homeostasis through regulators, such as Zur, AdcR, and ZntR. *Riemerella anatipestifer* is a significant *Flavobacteriales* pathogen causing acute serositis in ducks and other birds. In this study, we identified a homolog of ZntR, a regulator for zinc homeostasis, and demonstrated its contribution to the pathogenicity of *R. anatipestifer*. Deletion of *zntR* makes the bacteria hypersensitive to excess Zn^2+^ but not to other metals like manganese (Mn^2+^), copper (Cu^2+^), cobalt (Co^2+^), and nickel (Ni^2+^). Deletion of *zntR* also leads to intracellular zinc accumulation but not of other metals. Additionally, compared to the wild type, the deletion of *zntR* increases resistance to oxidants hydrogen peroxide (H_2_O_2_) and sodium hypochlorite (NaOCl), respectively. The deletion of *zntR* causes significant changes in transcriptional and protein expression levels, revealing 35 genes with potential zinc metabolism functions. Among them, *zupT*, which is inhibited by ZntR, is required for zinc transport and resistance to oxidative stress. Finally, deletion of *zntR* leads to attenuation of colonization in ducklings. In summary, ZntR is a crucial regulator for zinc homeostasis and contributes to the pathogenicity of *R. anatipestifer*.

**IMPORTANCE:**

Zinc homeostasis plays a critical role in the environmental adaptability of bacteria. *Riemerella anatipestifer* is a significant pathogen in poultry with the potential to encounter zinc-deficient or zinc-excess environment. The mechanism of zinc homeostasis in this bacterium remains largely unexplored. In this study, we showed that the transcriptional regulator ZntR of *R. anatipestifer* is critical for zinc homeostasis by altering the transcription and expression of a number of genes. Importantly, ZntR inhibits the transcription of zinc transporter ZupT and contributes to colonization in *R. anatipestifer*. The results are significant for understanding zinc homeostasis and the pathogenic mechanisms in *R. anatipestifer*.

## INTRODUCTION

Zinc is one of the most important transition metals in prokaryotes and eukaryotes (1). It is essential for key biochemical processes, such as DNA replication, repair, transcriptional regulation, and protein degradation (2, 3). Vertebrate hosts have evolved mechanisms to limit zinc availability and hinder bacterial growth, a defense known as nutritional immunity (4, 5). For example, neutrophils secrete calprotectin, a heterodimer of S100A8 and S100A9 proteins, which chelates zinc and other divalent cations during infection (6). Additionally, the Zn^2+^ transporter ZIP8, expressed in human macrophages, binds to the lysosomal protein Lamp1, reducing lysosomal Zn^2+^ and inhibiting bacterial growth (7).

Bacteria have evolved various zinc uptake systems to compete with the host for essential Zn^2+^. In gram-negative bacteria, zinc transport from the extracellular environment to the periplasm occurs via non-specific porins or TonB-dependent receptors, such as ZnuD in *Neisseria meningitidis* (8, 9). Under zinc-deficient conditions, the ZnuABC system, a high-affinity zinc transporter in *Salmonella typhimurium* and other bacteria, mediates zinc transport from the periplasm to the cytoplasm (10–12). Other identified ABC transporters involved in zinc uptake include AdcABC in *Streptococcus mutans* (13) and TroCBA in *Agrobacterium tumefaciens* (14). When zinc is abundant, some bacteria express the low-affinity zinc transporter ZupT, a member of the ZIP family, for cellular zinc import (15, 16). Although zinc is vital for bacterial survival and growth, excess zinc can cause toxic effects. Excessive zinc binds to non-homologous metal proteins and can even bind to metal-free proteins, leading to protein dysfunction, a phenomenon referred to as protein mismetallation (17). Zinc toxicity in bacteria impairs manganese uptake, oxidative stress resistance (18), central carbon metabolism (19), biofilm synthesis (20), and antibiotic resistance (21). Studies have shown that zinc can be used by the host as an antibacterial agent against pathogens. For example, the content of zinc in human macrophages infected with *Mycobacterium tuberculosis* increased over time and co-localized with *M. tuberculosis*, and the survival rate of zinc efflux protein deletion strains in macrophages was reduced (22). To avoid zinc toxicity, bacteria use zinc efflux mechanisms, including the P-type ATPase ZntA in *Vibrio parahaemolyticus* (23), the resistance-nodulation-division (RND) system CzcCBA in *Acinetobacter baumannii* (24), the cation diffusion facilitator (CDF) transporter CzcD in *Streptococcus pneumoniae* (25), ZitB in *S. typhimurium* (26), and YiiP in *Escherichia coli* (27).

Bacteria sense changes in zinc levels inside the cytoplasm through transcriptional regulators to regulate the expression of zinc uptake protein and zinc efflux protein, thus maintaining zinc homeostasis (28). Notable examples include Zur in *Yersinia pseudotuberculosis* (29), AdcR in *Streptococcus suis* (30), SczA in *S. pneumoniae* (25), SmtB in *Mycobacterium smegmatis* (31), and ZntR in *V. parahaemolyticus* (23). ZntR, a MerR family member, acts as a Zn^2+^-responsive transcriptional regulator of ZntA in *E. coli* (32). In *Y. pseudotuberculosis*, ZntR positively regulates the type VI secretion system (T6SS), maintaining zinc homeostasis and preventing bacterial death (33). In gram-positive *Staphylococcus aureus*, ZntR functions as a trans-repressor, binding to the *znt* promoter region to inhibit transcription of the *znt* zinc resistance operon (34).

*R. anatipestifer* is a gram-negative bacterium within the *Flavobacteriia* class, *Flavobacteriales* order, and *Weeksellaceae* family (35), and it is known to cause infections in ducks and other waterfowl (36). Infected ducks develop sepsis and infectious serositis (37). Twenty-one distinct serotypes of *R. anatipestifer* have been identified, with no observed cross-protection among them (36). Additionally, *R. anatipestifer* is multi-antibiotics resistant (38, 39), thereby complicating prevention and treatment with vaccines and other antibiotics. Our previous research identified genes involved in iron and manganese metabolism in *R. anatipestifer*, including ferric uptake regulator Fur (40), iron uptake protein FeoAB (41), iron efflux protein IetA (38), and manganese efflux proteins MetA, MetB (42), and TerC (43). However, genes responsible for zinc homeostasis have not yet been studied.

In this study, we identified the *B739_RS08595* gene of the *R. anatipestifer* CH-1 strain (RA CH-1) as a MerR family transcription regulator in the NCBI database, with 21.19% identity to ZntR in *E. coli* K-12. Various methodologies, including gene knockout, metal sensitivity assays, intracellular metal content analysis, and transcriptomic and proteomic evaluations, were used to explore the mechanisms of zinc homeostasis regulated by *B739_RS08595*. These results revealed that *B739_RS08595*, now designated *zntR*, is essential for zinc homeostasis and contributes to the colonization of RA CH-1.

## MATERIALS AND METHODS

### Bacterial strains, plasmids, primers, growth media, and antibiotics

Bacterial strains and plasmids are listed in Table S1. The primers are listed in Table S2. *E. coli* was grown on LB (Luria-Bertani) agar plates or in LB broth at 37°C. *R. anatipestifer* was grown on sheep blood plates or in TSB (tryptic soy broth) at 37°C. When necessary, the following antibiotics were added: for *E. coli*, ampicillin (Amp) at 100 µg/mL; and for *R. anatipestifer*, spectinomycin (Spc) at 80 µg/mL, polymyxin B (PB) at 128 µg/mL, cefoxitin (Cfx) at 1 µg/mL, and kanamycin (Kan) at 50 µg/mL.

### Construction of the mutant and complemented strains

The mutant strains were constructed as follows. Briefly, approximately 1,200 bp upstream and 1,200 bp downstream fragments of the *zntR* gene were amplified from the genomic DNA of RA CH-1. The spectinomycin resistance gene was amplified from the plasmid pAM238. We then used overlap PCR to fuse these three fragments to construct the Up-Spc-Down fusion fragment. Subsequently, this fragment was then introduced into the RA CH-1 strain via natural transformation (44). Finally, we screened the recombinant mutant strains on blood plates containing Spc and confirmed by PCR, resulting in the *zntR* deletion strain RA CH-1∆*zntR::spc*.

The markerless mutant strain RA CH-1∆*zupT* was constructed as follows. Briefly, approximately 800 bp upstream and 800 bp downstream fragments of the *zupT* gene were amplified from the genomic DNA of RA CH-1. We then cloned these fragments into the pBAD24::*cfx-sacB* plasmid (45). Subsequently, we amplified the Up-Cfx-sacB-Down fragment from the above-constructed plasmid and introduced it into the RA CH-1 strain via natural transformation. The recombinant strains were screened on blood plates containing Cfx and confirmed by PCR, resulting in the strain RA CH-1∆*zupT::cfx-sacB*. To obtain the second recombinant, we amplified approximately 1,900 bp upstream and 1,800 bp downstream fragments of *zupT* from the genomic DNA of RA CH-1, fused these two fragments using overlap PCR to create the Up-Down fusion fragment, and introduced this fusion fragment into the RA CH-1∆*zupT::cfx-sacB* strain via natural transformation. Finally, the recombinant markerless mutant strains were screened on blood plates containing 15% sucrose, confirmed by PCR, and named RA CH-1∆*zupT*.

The complemented strains were constructed as follows. Briefly, the coding sequences of *zntR* and *zupT* were amplified from the RA CH-1 genome and cloned into the pLMF02 plasmid, respectively. Then, the recombinant plasmid was introduced into the RA CH-1∆*zntR::spc* or RA CH-1∆*zupT* strains via conjugation (46). Finally, the complemented strains were screened on blood plates containing Cfx and Kan, confirmed by PCR, and designated as RA CH-1∆*zntR::spc* pLMF02*::zntR* (*ΔzntR^C^*) or RA CH-1∆*zupT* pLMF02*::zupT* (*ΔzupT^C^*).

### Growth curve

RA CH-1 pLMF02 (wild type, WT), RA CH-1∆*zntR::spc* pLMF02 (*ΔzntR*)*, ΔzntR^C^*, RA CH-1∆*zupT* pLMF02 (Δ*zupT*), and Δ*zupT*^C^ strains were cultured in 20 mL of TSB with or without 0.01 mM ZnSO_4_ supplementation, with shaking at 180 rpm, starting from an OD_600_ of 0.1 at 37°C to determine the growth rates within 12 h. Each experiment was performed in triplicate.

### Disk diffusion assay

Briefly, the WT, *ΔzntR,* and *ΔzntR^C^* strains were cultured at 37°C in TSB to an OD_600_ of ~1.5 and diluted to OD_600_ = 0.4. A total of 5 mL of melted TSA containing 0.75% agar and 100 µL of the bacterial solution were mixed and spread on TSA plates. Filter paper disks, each 6 mm in diameter, were placed on the plates, and 10 µL of the metal solution was applied to each disk (42). The metal solutions used were 1 M MnCl_2_, 0.4 M ZnSO_4_, 0.2 M CuCl_2_, 0.2 M CoCl_2_, and 1 M NiCl_2_. After incubating at 37°C for 12 h, the inhibition zones were measured. All experiments were performed in triplicate.

### Inductively coupled plasma-mass spectrometry

Briefly, the WT, *ΔzntR, ΔzntR^C^*, Δ*zupT*, and Δ*zupT*^C^ strains were cultured in TSB or TSB supplemented with 0.05 mM MnCl_2_, 0.2 mM ZnSO_4_, 0.02 mM CuCl_2_, 0.15 mM FeSO_4_, 0.1 mM CoCl_2_, 1 mM NiCl_2_ or 0.01 mM ZnSO_4_, respectively, to an OD_600_ of ~1.5. The bacterial cells were washed with PBS containing 0.1 M EDTA and resuspended with ultrapure water. The bacterial cells were sonicated, and the lysates were centrifuged to collect the supernatant. Protein content was quantified using a NanoDrop 2000, and the supernatants were incubated at −20°C for 12 h. A total of 400 µL of supernatants was then added with 600 µL of ultrapure water (containing 0.1% Triton X-100 and 5% HNO_3_). After incubating at 95°C for 30 min, the lysates were centrifuged, and the supernatant was collected and then diluted with 2% HNO_3_ to 5 mL to determine the metal content (42). The samples were then sent to Sichuan Shanshi Technology Co., Ltd. for inductively coupled plasma-mass spectrometry (ICP-MS) (Elan DRC II, PerkinElmer). Gallium was used as the internal standard during ICP-MS detection. Each experiment was performed in triplicate.

### Oxidative stress sensitivity assay

Briefly, the WT, *ΔzntR, ΔzntR^C^*, Δ*zupT*, and Δ*zupT*^C^ strains were cultured at 37°C in TSB to an OD_600_ of ~1.5 and diluted to OD_600_ = 0.5. A 1 mL aliquot of each bacterial suspension was treated with either 8 mM H_2_O_2_ or 0.25% NaOCl (42). After incubation at 37°C for 30 min, the bacterial cells were washed three times. The bacterial solution was diluted and spread on sheep blood plates to determine CFU counts and survival rates. All experiments were repeated at least three times.

### Virulence and colonization assays

For the virulence assay, 1-day-old ducklings were adaptively raised until 3 days old, with 10 ducklings per group. The WT and *ΔzntR* strains were cultured at 37°C in TSB to an OD_600_ of ~1.5 and adjusted to OD_600_ = 2.5 using PBS. Each duckling was intramuscularly injected with 200 µL of bacterial suspension into the legs (10^9^ CFU per duck), while the control group was injected with 200 µL of PBS. The ducklings were monitored for signs of dying symptoms, and those showing severe symptoms were euthanized via forced CO_2_ inhalation. Mortality was observed and recorded daily for 7 days.

For the colonization assay, six ducklings were each injected with the WT and *ΔzntR* at a dose of 10^9^ CFUs. At 24 h post-infection, three random living ducklings were selected from each group for euthanasia. A total of 0.1 g of tissue, including blood, liver, spleen, and brain was collected in 0.9 mL PBS. The solutions were ground, and the homogenized solutions were diluted and spread on blood plates containing Kan and PB to determine CFU counts.

### Transcriptome analysis

For RNA extraction, the WT and *ΔzntR* strains were cultured at 37°C in TSB to an OD_600_ of ~1.5 and adjusted to OD_600_ = 0.5 using TSB. Total RNA was extracted from the bacterial pellet using an RNAprep Pure Cell/Bacteria Kit (catalog number: DP430; Beijing Tiangen Biotech Co., Ltd.). We used a NanoDrop 2000 to assess the concentration and quality of RNA samples and then sent the samples to Shanghai Majorbio Bio-Pharm Technology Co., Ltd. for RNA sequencing (RNA-seq). Library preparation for RNA transcriptome sequencing was performed using the TruSeq Stranded Total RNA Library Prep Kit. The rRNA was first removed from the total RNA, and the mRNA was cleaved into ~200 bp fragments using fragmentation buffer. Single-stranded cDNA was synthesized with random primers, and the second-strand cDNA was synthesized with dUTP instead of dTTP. The ends of double-stranded cDNA were blunted with the End Repair Mix and an A base was added at the 3′ end. The cDNA was then digested with the UNG enzyme to remove the second strand. Finally, the remaining first-strand cDNA was quantified using Qubit 4.0 and sequenced on the NovaSeq XPlus platform to obtain raw data. The raw data were quality controlled using fastp, Sickle, and SeqPrep software and then assembled using Cufflinks software. The quality-controlled data (clean reads) was aligned with the *R. anatipestifer* CH-1 genome (GCA_000295655.1) to obtain read counts using Bowtie2 and RSeQC software. The reference genome was compared with Clusters of Orthologous Groups of proteins (COG), Gene Ontology (GO), Kyoto Encyclopedia of Genes and Genomes (KEGG), and other databases to obtain the functional annotations of the genes. Differential expression analysis between samples was performed using DEGseq software with the following screening criteria: *P* ≤ 0.05 and fold change ≥ 2.

### Proteome analysis

The WT and *ΔzntR* were cultured at 37°C in TSB to an OD_600_ of ~1.5, with each strain prepared in triplicate. All the medium was discarded, and the bacterial cells were incubated in liquid nitrogen for 20 min and then sent to Shanghai Majorbio Bio-Pharm Technology Co., Ltd. for further processing. To extract total protein, the samples were ground for 2 min after the addition of protein lysate and lysed on ice for 30 min. Protein content was quantified using the BCA method, and protein quality was assessed using SDS-PAGE. Qualified protein samples were subjected to reductive alkylation and trypsin digestion to prepare polypeptides. After desalting and quantifying the peptide samples, an equal amount of each sample was subjected to data-independent acquisition mass spectrometry using an Astral mass spectrometer (Thermo, USA). Briefly, the uPAC High Throughput column (Thermo, USA) was used with solvent A (water containing 2% ACN and 0.1% formic acid) and solvent B (water containing 80% ACN and 0.1% formic acid). The chromatography run time was set to 8 min. Detection was performed over a mass range of 380–980 *m*/*z* (MS1) and 150–2,000 *m*/*z* (MS2). Qualitative and quantitative analyses were performed using Spectronaut 18 software. The parameter settings were as follows: peptide length range of 7–45; trypsin/P enzyme cleavage; maximum missed cleavage sites set to 2; cysteine formamide methylation as a fixed modification; and methionine oxidation and protein N-terminal acetylation as variable modifications. Proteins with more than 90% missing data were removed, and matrices with a small number of missing values (no more than one missing value among three replicates) were filled using the SeqKNN method. All proteins identified by mass spectrometry were compared with COG, GO, KEGG, and other databases to obtain functional information. Differential expression analysis between samples was performed using Student’s *t*-test. Some proteins were detected exclusively in WT or *zntR* deletion strains. In our analysis, when the denominator is zero, the fold change value is set to twice the maximum fold change value of all samples when the molecular denominator is not zero. When the molecule is zero, the fold change value is assigned a fixed value of 0.00001.

### qRT-PCR

Total RNA from WT and Δ*zntR* was extracted as described in Transcriptome analysis. A total of 900 ng of RNA was reverse transcribed and served as the template for qRT-PCR using SYBR Green I chimeric fluorescence reagent (Nanjing Vazyme Biotech Co., Ltd.) to obtain the Ct values of the target genes. With 16S rRNA as the internal reference gene, we used the 2^−ΔΔCt^ method to calculate gene expression levels (41).

### Spot assay

The WT, *ΔzupT*, and *ΔzupT^C^* were cultured at 37°C in TSB to an OD_600_ of ~1.5 and adjusted to OD_600_ = 0.5 using PBS. We serially diluted the bacterial solution to 10^−6^ and then dropped 10 μL of each dilution onto TSA or TSA supplemented with 0.01 mM MnCl_2_, 0.01 mM ZnSO_4_, 0.01 mM CuCl_2_, 0.01 mM FeSO_4_, 0.01 mM CoCl_2_, or 0.01 mM NiCl_2_. After culturing for 12–24 h at 37°C, all the plates were photographed. All the experiments were repeated at least three times.

### Data analysis

Proteome and transcriptome analyses were performed using the Majorbio Cloud Platform (www.majorbio.com) and the OmicShare tool (https://www.omicshare.com). Data processing and statistical analysis were conducted using GraphPad Prism 8. Statistical significance was determined using one-way ANOVA, Mantel-Cox test, and the *t*-test, with significance indicated by asterisks (*P < 0.05; **P < 0.01; ***P < 0.005; and ****P < 0.001). “ns” denotes no significant difference.

## RESULTS

### Deletion of *B739_RS08595* leads to hypersensitivity to excess zinc in RA CH-1

**Fig 1:**
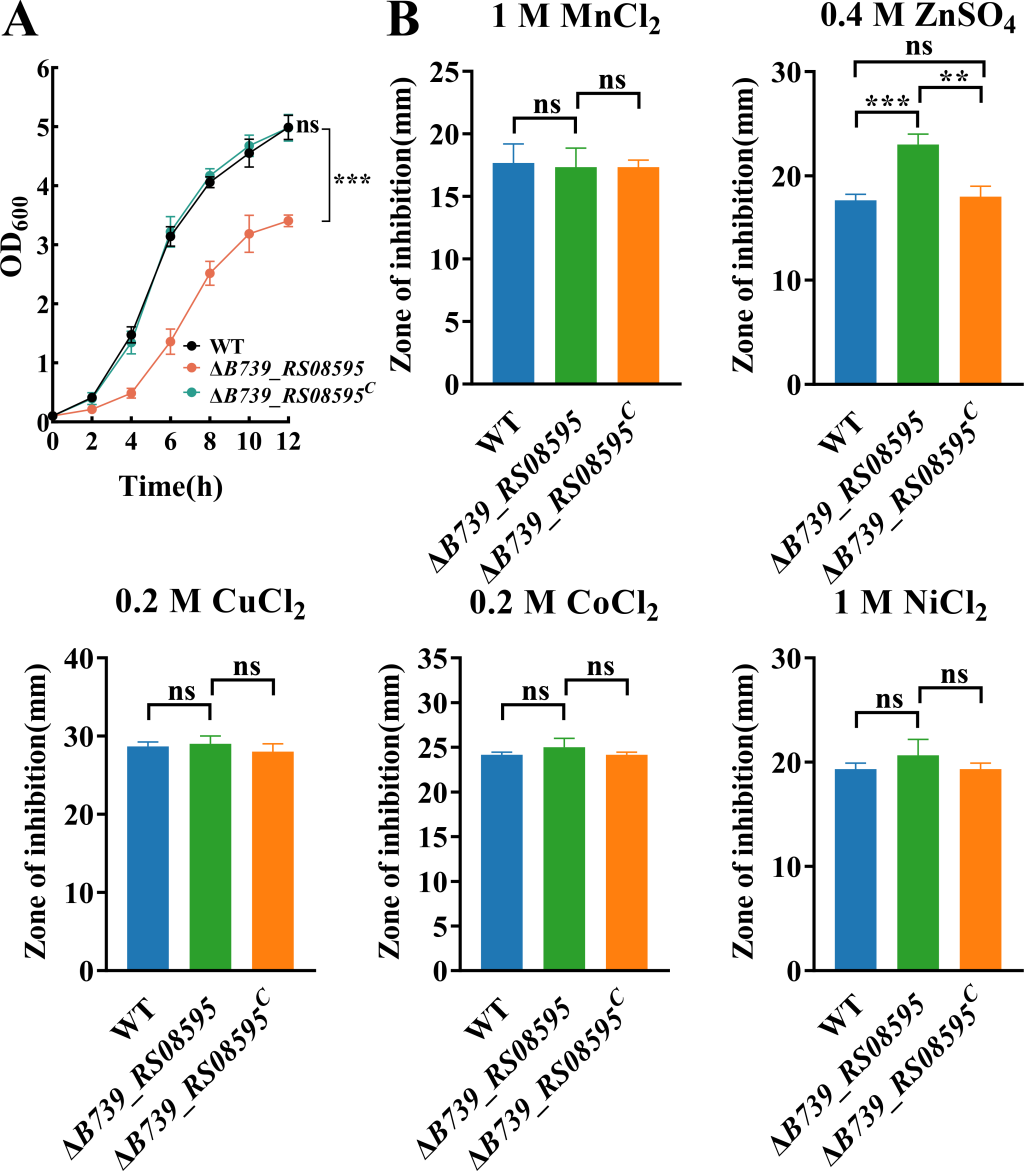
The *∆B739_RS08595* is more sensitive to excess zinc than the WT. (A) The growth curve of RA CH-1 pLMF02 (WT), RA CH-1 *∆B739_RS08595* pLMF02 (*∆B739_RS08595*), and RA CH-1 *∆B739_RS08595* pLMF02*::B739_RS08595* (*∆B739_RS08595*^C^) in TSB. (**B)** The sensitivity of the WT, *∆B739_RS08595*, and *∆B739_RS08595^C^* to different metals was measured by disk diffusion assay. The WT, *∆B739_RS08595*, and *∆B739_RS08595^C^* were grown to the exponential phase in TSB. A volume of 100 µL of bacteria was mixed with 4 mL of TSB with 0.75% soft agar and then poured onto TSB agar plates. Filter paper disks (6 mm) were placed on top of the agar, and 10 µL of 1 M MnCl_2_, 0.4 M ZnSO_4_, 0.2 M CuCl_2_, 0.2 M CoCl_2_, and 1 M NiCl_2_ were added to the disks, respectively. Growth inhibition zones (in mm) around the discs were measured after 24 h incubation at 37°C. Data represent means and standard deviations of results from three independent experiments. Significant differences compared to the WT were determined by one-way ANOVA. The *P* values of the data were calculated by one-way ANOVA using GraphPad Prism 8. Asterisks denote significant differences (**P＜0.01; ***P < 0.005). ns, no significant difference.

The *B739_RS08595* gene of RA CH-1 is annotated as a MerR family transcription regulator in the NCBI database. Sequence analysis shows that the protein encoded by *B739_RS08595* shares 21.19% identity with *E. coli* ZntR (32), 21.13% identity with *V. parahaemolyticus* ZntR (23), 19.87% identity with *Y. pseudotuberculosis* ZntR (33), 19.59% identity with *Brucella abortus* ZntR (47), 17.69% identity with *A. tumefaciens* ZntR (48), 13.64% identity with *Cupriavidus metallidurans* ZntR (49), and 10.79% identity with *S. aureus* ZntR (34) (Fig. S1). To determine the role of *B739_RS08595* in RA CH-1, we constructed the *B739_RS08595* deletion strain and its complement strains. Growth assessment showed that compared with the WT, the *B739_RS08595* deletion strain exhibited significant growth impairment in TSB and that it was restored by the expression of *B739_RS08595* in the mutant strain in *trans* (Fig. 1A). We then evaluated metal homeostasis involvement by testing sensitivity to MnCl_2_, ZnSO_4_, CuCl_2_, CoCl_2_, and NiCl_2_ using a disk diffusion assay. The result showed that *B739_RS08595* deletion strain had increased sensitivity to ZnSO_4_ but not to other metals (MnCl_2_, CuCl_2_, CoCl_2_, and NiCl_2_) compared to that of the wild type (Fig. 1B), indicating *B739_RS08595’s* specific role in zinc homeostasis.

### Deletion of *zntR* (*B739_RS08595*) increased intracellular zinc content in RA CH-1

**Fig 2:**
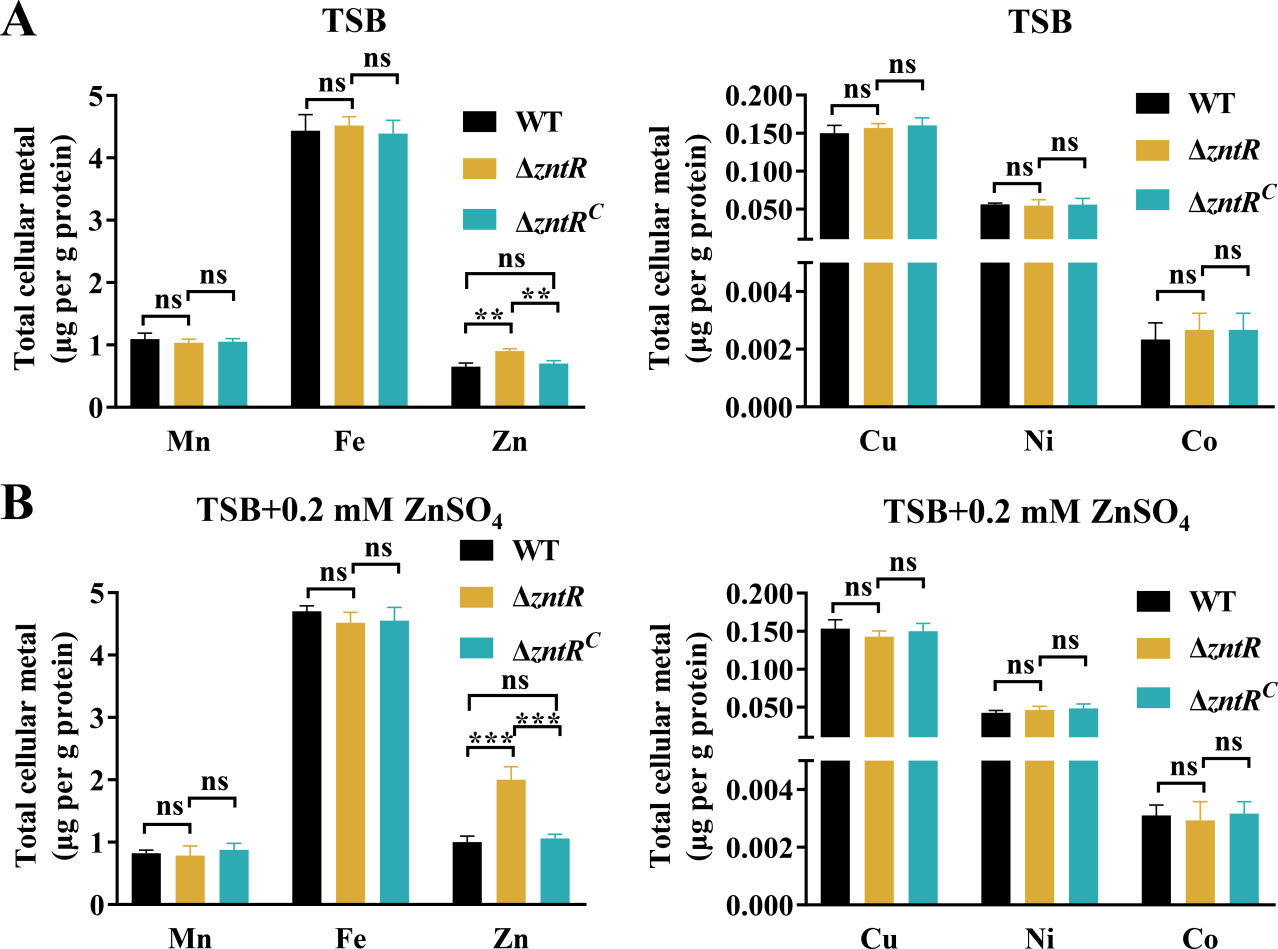
The deletion of *zntR* leads to the accumulation of zinc in the bacterial cell. (A) Intracellular Mn, Zn, Cu, Fe, Co, and Ni content of WT, Δ*zntR*, and Δ*zntR^C^* in TSB was measured using ICP-MS. (**B)** Intracellular Mn, Zn, Cu, Fe, Co, and Ni content of WT, Δ*zntR*, and Δ*zntR^C^* in TSB supplemented with 0.2 mM ZnSO_4_ was measured using ICP-MS. The results are expressed as micrograms of metal content per gram of protein. Data represent means and standard deviations of results from three independent experiments. Significant differences compared to the WT were determined by one-way ANOVA. The *P* values of the data were calculated by one-way ANOVA using GraphPad Prism 8. Asterisks denote significant differences (**P＜0.01 and ***P < 0.005) between two groups. ns, no significant difference.

The increased sensitivity of the *B739_RS08595* deletion strain to excessive zinc may result from intracellular zinc accumulation. To test this hypothesis, we measured the intracellular content of six metals in the WT, the *B739_RS08595* deletion strain, and its complement strains grown in TSB or TSB containing 0.2 mM ZnSO_4_ using ICP-MS. As shown in Fig. 2A, the *B739_RS08595* deletion strain had significantly higher intracellular zinc in TSB compared to the WT, with a more pronounced increase under zinc supplementation (Fig. 2B). There were no notable differences in other metal contents between the WT and the *B739_RS08595* deletion strain under normal conditions (Fig. 2A) or under excess metal conditions (Fig. S3). These findings suggest that elevated intracellular zinc levels in the *B739_RS08595* deletion strain account for its increased sensitivity to excessive zinc. Based on these findings and the data described below, we renamed *B739_RS08595* as ZntR (Zn(II)-responsive transcriptional regulator).

### Deletion of *zntR* leads to increased resistance to H_2_O_2_ and NaOCl

**Fig 3:**
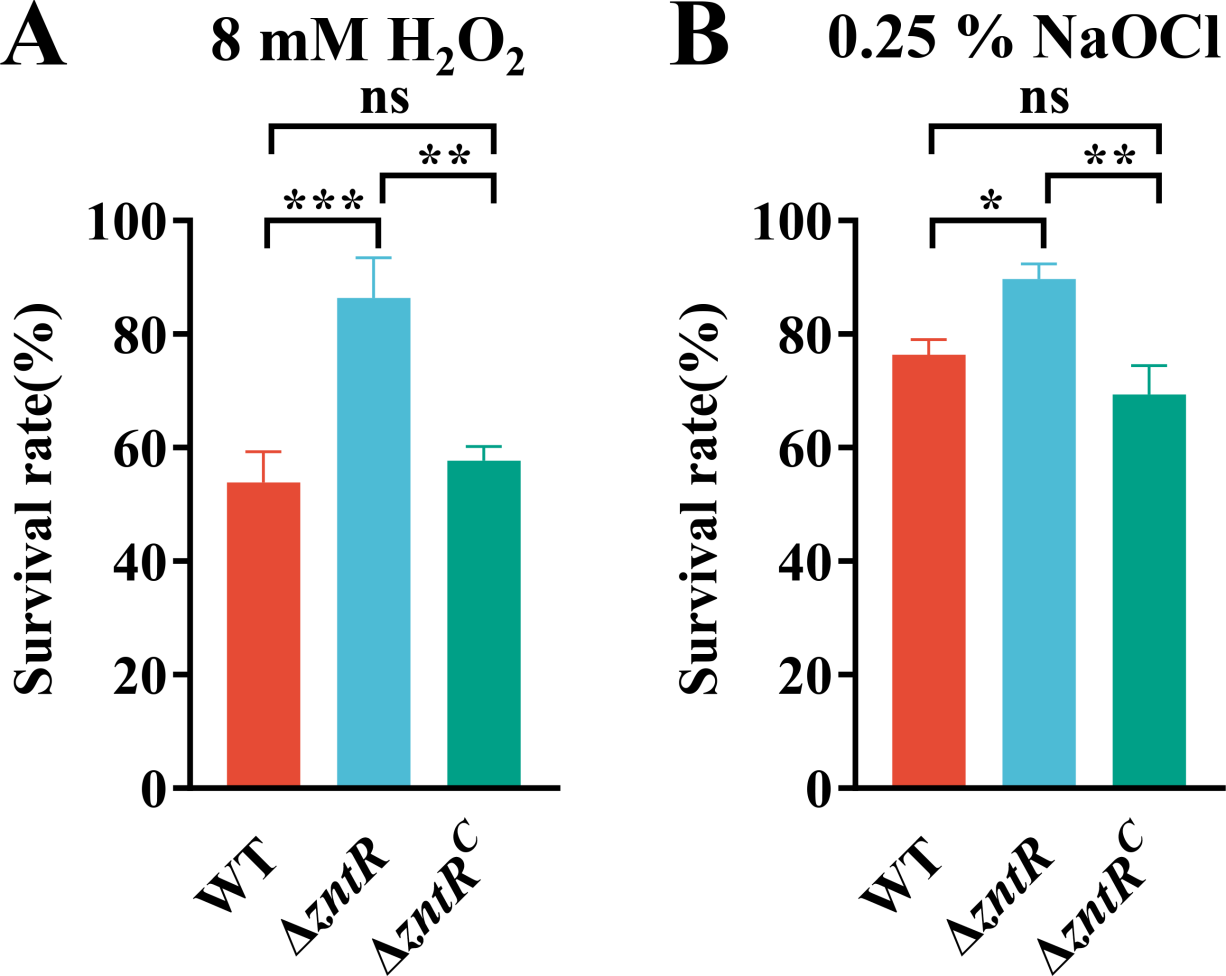
The *∆zntR* is more resistant to H_2_O_2_ and NaOCl than the WT. (A) The sensitivity of the WT, *∆zntR*, and *∆zntR^C^* to 8 mM H_2_O_2_. (**B)** The sensitivity of the WT, *∆zntR*, and *∆zntR^C^* to 0.25% NaOCl. The WT, *∆zntR*, and *∆zntR^C^* were grown to the exponential phase in TSB. The bacterial solution was incubated with 8 mM H_2_O_2_ or 0.25% NaOCl. After incubation at 37°C for 30 min, the bacterial solutions were diluted serially and then spread onto sheep blood plates for CFU counting and survival rate calculation. Data represent means and standard deviations of results from three independent experiments. Significant differences compared to the WT were determined by one-way ANOVA. The *P* values of the data were calculated by one-way ANOVA using GraphPad Prism 8. Asterisks denote significant differences (*P＜0.05; **P＜0.01; and ***P < 0.005) between two groups.

Bacterial metal homeostasis is crucial for oxidative stress resistance. Zinc can competitively inhibit iron, thereby reducing the reactive oxygen species (ROS) generated by iron in the Fenton reaction (50). The deletion of *zntR* disrupted zinc homeostasis in *R. anatipestifer*, prompting an investigation into its impact on oxidative stress resistance. We measured the sensitivity of WT, Δ*zntR*, and Δ*zntR*^C^ to H_2_O_2_ and NaOCl, respectively. As shown in Fig. 3, Δ*zntR* exhibited a significantly higher survival rate than WT and Δ*zntR*^C^ after exposure to 8 mM H_2_O_2_ or 0.25% NaOCl at 37℃ for 30 min. These results suggest that ZntR is involved in the oxidative stress response in *R. anatipestifer*.

### Deletion of *zntR* leads to dramatic changes in the transcriptome

**Fig 4:**
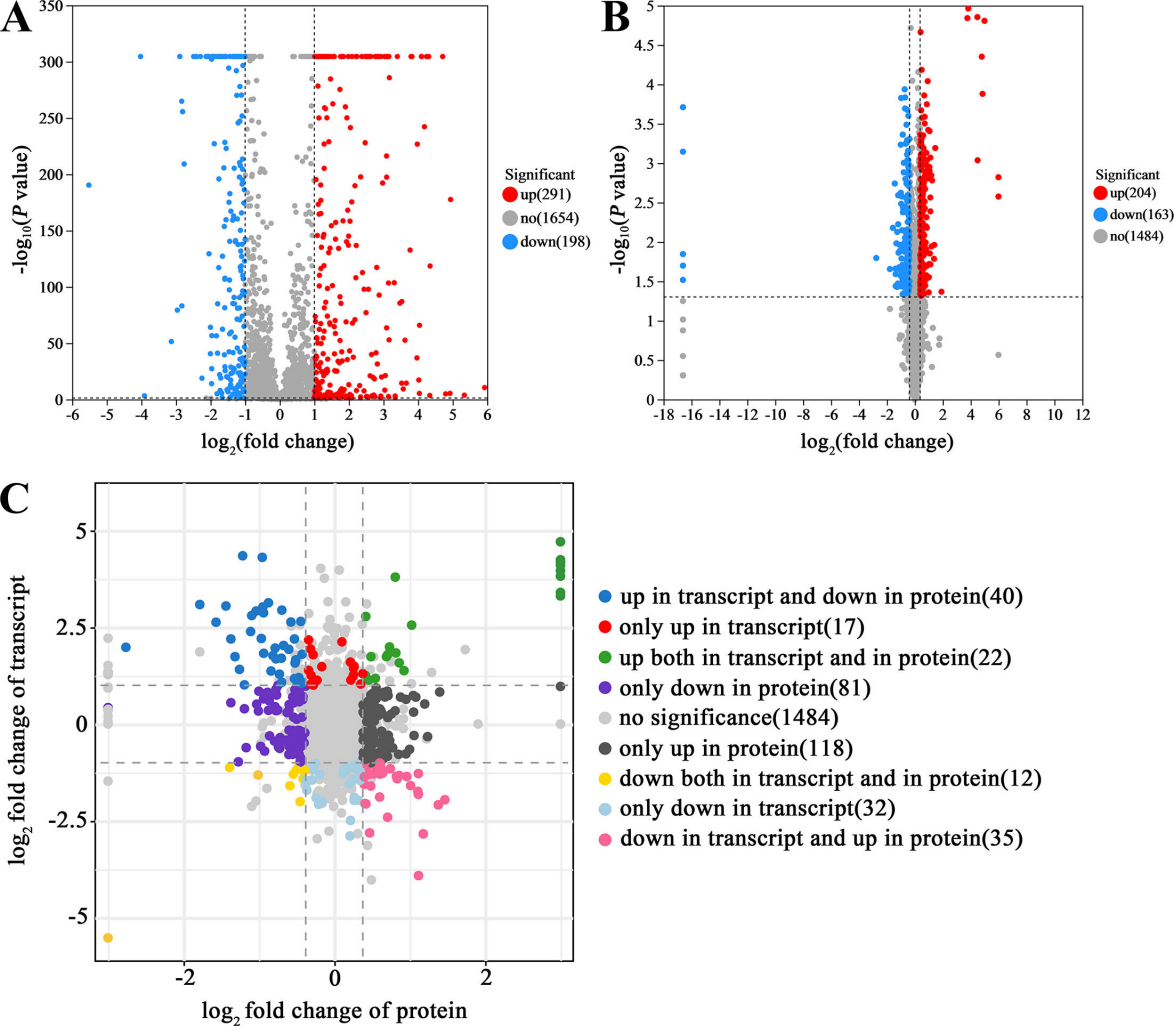
The transcriptome and proteome of WT and Δ*zntR,* as well as the combined analysis. (A) Volcano map of differentially expressed genes of Δ*zntR* compared with the WT in the transcriptome. (**B)** Volcano map of differentially abundant proteins of Δ*zntR* compared with the WT in the proteome. Red dots represent upregulated genes. Blue dots represent downregulated genes. Gray dots represent no-regulated genes. (**C)** Combined analysis of proteome and transcriptome. A total of 1,841 genes were identified in the intersection of the transcriptome and proteome, of which 662 genes had *P* values less than 0.05 in both analyses. Among them, 175 genes were upregulated, and 133 genes were downregulated in the proteome, and 79 genes were upregulated and 79 genes were downregulated in the transcriptome. Some genes exhibit identical *P* values or log_2_FC values, which is a result of their significant and consistent differential expression.

The above experiments demonstrated ZntR’s role in maintaining zinc homeostasis in RA CH-1. To explore zinc metabolism mechanisms and identify ZntR-regulated genes, we performed transcriptome sequencing of WT and Δ*zntR*. As shown in Fig. 4A, compared with WT, Δ*zntR* had 291 upregulated genes and 198 downregulated genes when grown in TSB (*P* ≤ 0.05, fold change ≥ 2). Among the upregulated genes, 10 genes were annotated as TonB-dependent transporters related to metal transport, including *B739_RS00805*, *B739_RS00475*, *B739_RS05255*, *B739_RS00430*, *B739_RS01550*, *B739_RS01890*, *B739_RS06990*, *B739_RS00320*, *B739_RS04295*, and *B739_RS00540*; three genes were annotated as T9SS type A sorting domain-containing proteins, including *B739_RS00425*, *B739_RS06145*, and *B739_RS00445*; one gene, *B739_RS07625,* was annotated as a ZIP family metal transporter; and three genes encoding proteins were predicted to have zinc-binding ability, including *B739_RS00465*, *B739_RS09255*, and *B739_RS08645* (Table S3). Among the downregulated genes, six genes were annotated as TonB-dependent transporters, including *B739_RS02540*, *B739_RS03865*, *B739_RS05435*, *B739_RS02835*, *B739_RS02960*, and *B739_RS01970*; four genes were annotated as efflux RND transporter components, including *B739_RS08835*, *B739_RS08830*, *B739_RS08825*, and *B739_RS04275*; and two genes encoding proteins were predicted to have zinc-binding ability, including *B739_RS09300* and *B739_RS10930* (Table S3).

### Deletion of *zntR* also leads to dramatic changes in the proteome

To assess the impact of *zntR* deletion on the protein expression, we performed proteome mass spectrometry analyses of WT and Δ*zntR*. As shown in Fig. 4B, compared to the WT, Δ*zntR* had 204 upregulated proteins and 163 downregulated proteins (*P* ≤ 0.05, fold change ≥ 1.3). Among upregulated proteins, seven proteins were annotated as TonB-dependent receptors related to metal transportation, including B739_RS01470, B739_RS02540, B739_RS10310, B739_RS04255, B739_RS05365, B739_RS01235, and B739_RS10585 (Table S4); six proteins were annotated as cation/multidrug efflux pumps, including B739_RS08825, B739_RS08835, B739_RS08830, B739_RS10780, B739_RS03800, and B739_RS03805 (Table S4); one protein B739_RS01815 was annotated as a heavy metal-translocating P-type ATPase (17.91% identity with *E. coli* ZntA); and three proteins were predicted to have zinc-binding ability (Table S4). Among downregulated proteins, eight proteins were annotated as TonB-dependent receptors, including B739_RS02960, B739_RS06605, B739_RS00430, B739_RS00475, B739_RS05255, B739_RS01970, B739_RS00805, and B739_RS04305; and four proteins were predicted to have zinc-binding ability, including B739_RS09300, B739_RS05660, B739_RS03715, and B739_RS00465 (Table S4).

### Combined analysis of proteome and transcriptome

A total of 1,841 genes were identified at the intersection of the transcriptome and proteome, with 662 genes showing *P-*values less than 0.05 in both analyses. As shown in Fig. 4C, only 22 genes and their corresponding proteins were upregulated, while 12 genes and their corresponding proteins were downregulated, indicating intricate post-transcriptional or translational regulation in RA CH-1. Among these, the putative TonB-dependent receptors *B739_RS01970* and *B739_RS02960*, along with their corresponding proteins, were downregulated in *ΔzntR*. Conversely, *B739_RS08645,* annotated as an M14 family zinc carboxypeptidase, and its corresponding protein were upregulated in *ΔzntR* (Table S5).

### The gene *zupT*, which is inhibited by ZntR, is involved in zinc transport and contributes to oxidative stress resistance

**Fig 5:**
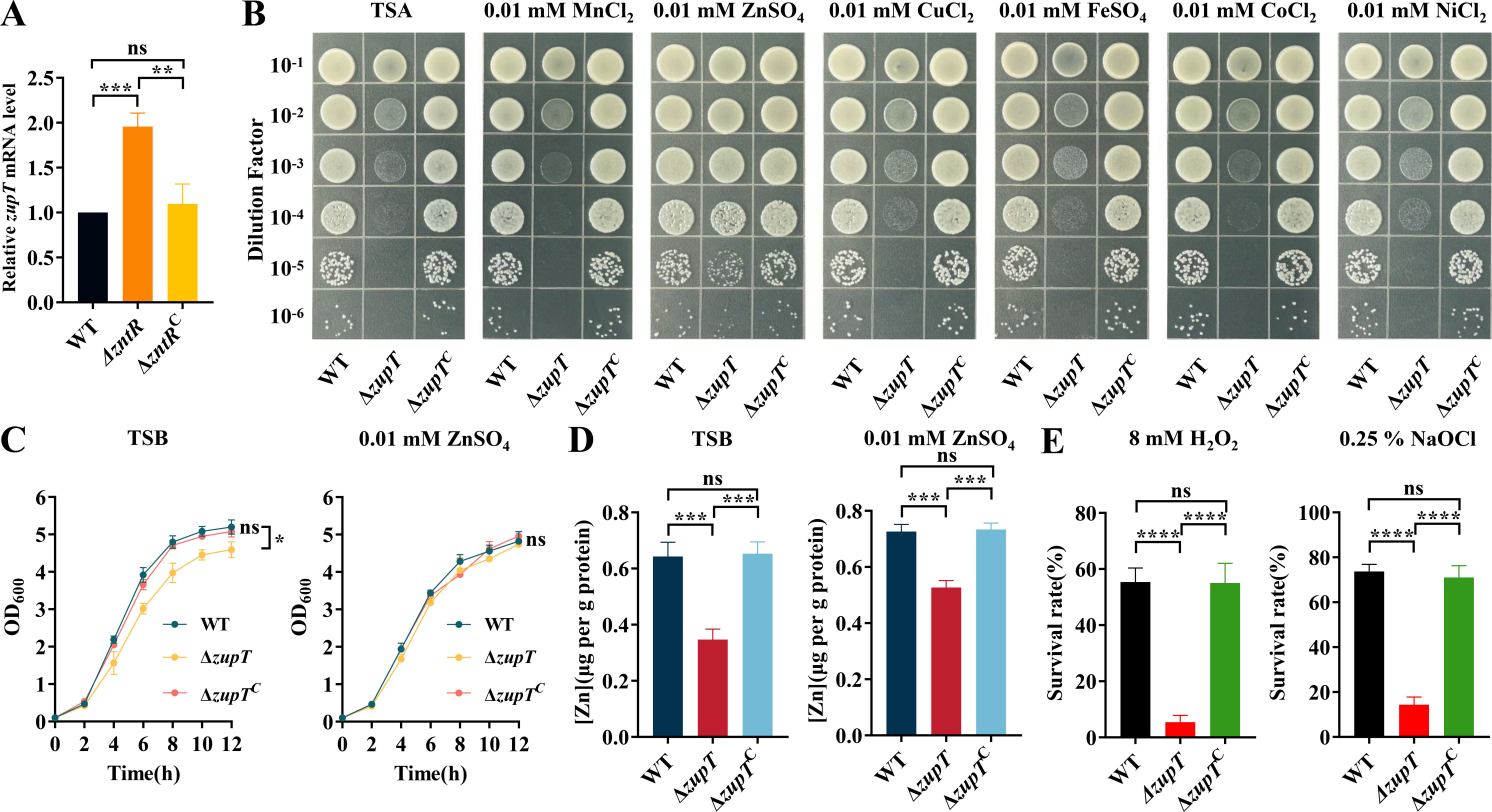
ZupT is involved in zinc uptake and oxidative stress resistance. (A) Relative *zupT* mRNA level of WT, Δ*zntR*, and Δ*zntR^C^* in TSB was measured using qRT-PCR. (**B)** The growth of WT, *∆zupT*, and *∆zupT*^C^ on TSA and TSA supplemented with 0.01 mM MnCl_2_, 0.01 mM ZnSO_4_, 0.01 mM CuCl_2_, 0.01 mM FeSO_4_, 0.01 mM CoCl_2_, and 0.01 mM NiCl_2_, respectively. The WT, *ΔzupT*, and *ΔzupT^C^* were grown in TSB at 37°C to the OD_600_ of ~1.5 and then adjusted to OD_600_ = 0.5 using PBS. The bacterial solution was serially diluted 10-fold in PBS to 10^−6^, and 10 µL of the solution was spotted onto TSA and TSA supplemented with 0.01 mM MnCl_2_, 0.01 mM ZnSO_4_, 0.01 mM CuCl_2_, 0.01 mM FeSO_4_, 0.01 mM CoCl_2_, and 0.01 mM NiCl_2_, respectively. The plates were incubated at 37°C overnight for photographing. The experiment was repeated three times, and representative spot assays for each condition are shown. (**C)** The growth curve of WT, *∆zupT*, and Δ*zntR^C^* in TSB and TSB supplemented with 0.01 mM ZnSO_4_. Significant differences compared to the WT were determined by one-way ANOVA. (**D)** Intracellular Zn content of WT, Δ*zupT*, and Δ*zupT^C^* in TSB and TSB supplemented with 0.01 mM ZnSO_4_ was measured using ICP-MS. (**E)** The sensitivity of the WT, *∆zupT*, and *∆zupT^C^* to 8 mM H_2_O_2_ and 0.25% NaOCl. Significant differences compared to the WT were determined by one-way ANOVA. The *P* values of the data were calculated by one-way ANOVA using GraphPad Prism 8. Asterisks denote significant differences (*P＜0.05; **P＜0.01; ***P < 0.005; and ****P < 0.001).

Transcriptome data revealed that *B739_RS07625*, a ZIP family metal transporter, was upregulated 2.3-fold in Δ*zntR* compared to the WT, suggesting ZntR inhibits its transcription. To verify this, qRT-PCR was performed to detect the transcript of *B739_RS07625* in WT, Δ*zntR*, and Δ*zntR*^C^. As shown in Fig. 5A*, B739_RS07625* was upregulated approximately twofold in Δ*zntR* relative to WT and Δ*zntR^C^*, consistent with the transcriptome data.

Sequence analysis showed that the protein encoded by *B739_RS07625* has low identity with well-characterized ZupT proteins: 19.77% identity with *E. coli* ZupT (51), 19.38% identity with *S. enterica* ZupT (16), 17.91% identity with *Clostridioides difficile* (*C. difficile*) ZupT (15), and 17.87% with *C. metallidurans* ZupT (52) (Fig. S2). To determine the function of *B739_RS07625* in RA CH-1, we constructed the *B739_RS07625* deletion strain and its complemented strain. We then assessed the impact of *B739_RS07625* depletion on zinc transportation. As shown in Fig. 5B, the *B739_RS07625* deletion strain exhibited significantly impaired growth on TSA plates compared with the WT and the complemented strain. Moreover, this growth impairment was able to be restored to WT growth levels with 0.01 mM ZnSO_4_ supplementation on the TSA plate, while MnCl_2_, CuCl_2_, FeSO_4_, CoCl_2_, or NiCl_2_ supplementation did not restore the growth of ∆*B739_RS07625*. Consistently, compared to the WT and the complemented strain, the growth ability of ∆*B739_RS07625* was significantly impaired in the TSB liquid medium, and the growth defect of ∆*B739_RS07625* was able to be restored through 0.01 mM ZnSO_4_ supplementation in the TSB (Fig. 5C). Based on these findings, we renamed B739_RS07625 as zinc uptake transporter (ZupT).

Next, we measured intracellular zinc content in WT, Δ*zupT*, and Δ*zupT*^C^ strains cultured in TSB or TSB containing 0.01 mM ZnSO_4_ using ICP-MS. As shown in Fig. 5D, Δ*zupT* had significantly lower intracellular zinc than the WT and the complemented strain when cultured in TSB (Fig. 5D). Similarly, zinc content of Δ*zupT* was also lower than that of the WT and the complemented strain when cultured in TSB supplemented with 0.01 mM ZnSO_4_ (Fig. 5D). These findings indicated that ZupT of RA CH-1 was specifically involved in zinc uptake.

Since zinc is a cofactor for superoxide dismutase, we also assessed the sensitivity of WT, Δ*zupT*, and Δ*zupT*^C^ to H_2_O_2_ and NaOCl, respectively. As shown in Fig. 5E, Δ*zupT* had a significantly lower survival rate than the WT and the complemented strain when exposed to 8 mM H_2_O_2_ or 0.25% NaOCl. These results indicate that ZupT functions as a zinc importer and is involved in oxidative stress resistance in *R. anatipestifer*.

### ZntR contributes to the pathogenicity in RA CH-1

**Fig 6:**
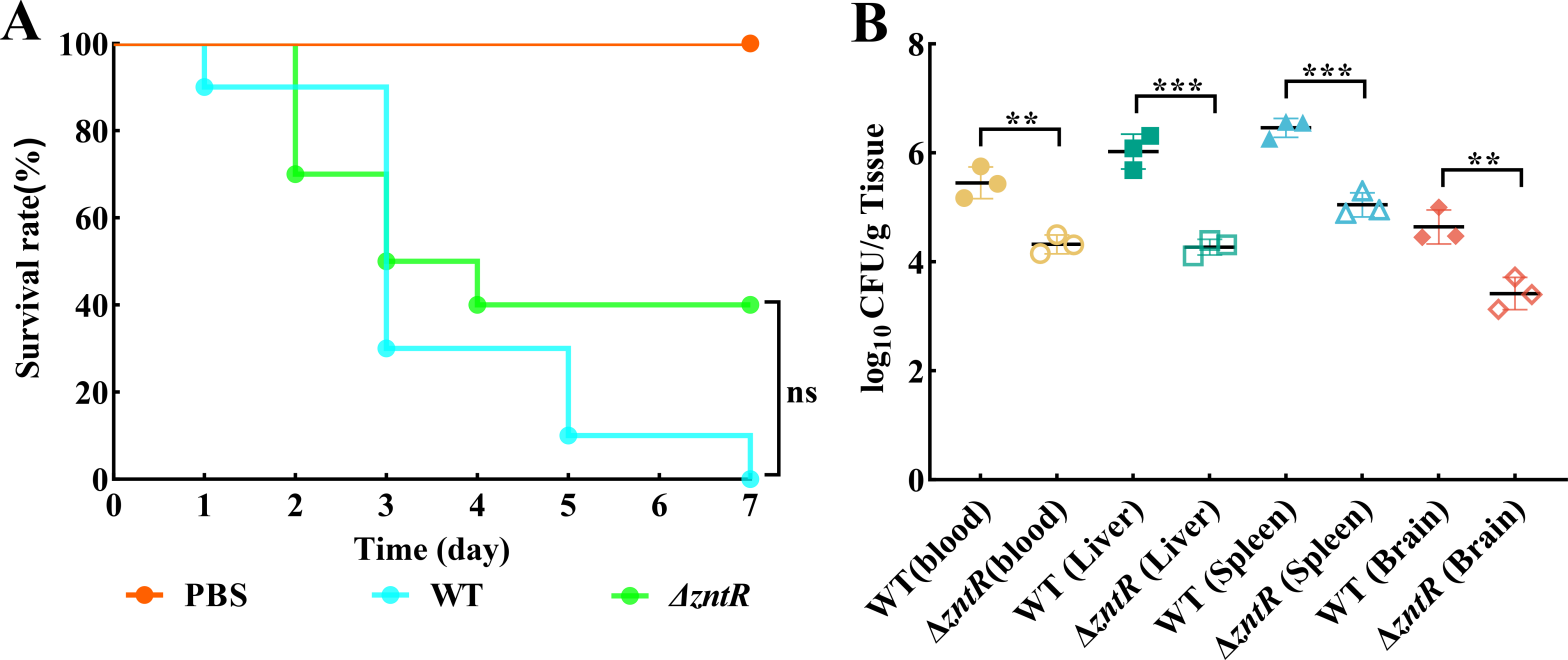
The *zntR* of *R. anatipestifer* CH-1 is required for the colonization in the duckling infection model. (A) Ten 3-day-old ducklings were injected with 10^9^ CFU of *R. anatipestifer* strains or PBS by the leg, and the survival rate of ducklings was detected every day within 7 days after the challenge. (**B)** Three 3-day-old ducklings were injected with 10^9^ CFU of *R. anatipestifer* strains or PBS by leg. At 24 h post-infection, *R. anatipestifer* was isolated from the blood, livers, spleens, and brains of ducklings. The data points represent the CFU/g values of the indicated organs in individual ducklings. The significant differences in virulence assays were determined by the Mantel-Cox test. The significant differences of colonization assays were determined by an unpaired *t*-test. The *P* values of the data were calculated using GraphPad Prism 8. Asterisks denote significant differences (**P＜0.01 and ***P < 0.005) between two groups. ns, no significant difference.

To investigate if *zntR* deletion has an effect on virulence, we examined its mortality and colonization in ducklings. The lethality rate of ducklings injected with the WT strain reached 100% within 7 days, whereas it decreased to 60% for those injected with the Δ*zntR* strain (Fig. 6A). As shown in Fig. 6B, Δ*zntR* exhibited significantly reduced tissue burdens in the heart, liver, spleen, and brain at 24 h post-infection compared to the WT. These results indicate that ZntR is important for the colonization of RA CH-1 in ducklings.

## DISCUSSION

Zinc is an essential element for the survival of all bacteria (5), serving as a cofactor for numerous proteins and playing critical roles in catalysis, regulation, and structural functions (53). Bacteria have evolved various zinc importers to secure sufficient zinc, primarily including the high-affinity zinc uptake system ZnuABC (12) and the low-affinity zinc uptake system ZupT (16). However, excessive zinc is toxic to bacteria (18, 19, 21). To prevent zinc toxicity, bacteria employ zinc efflux systems for detoxification. At present, at least three primary systems have been well-studied in different bacteria, including P-type ATPase, RND efflux pump, and CDF transporter (54). Bacteria detect changes in intracellular zinc concentration primarily via the transcriptional regulators Zur and ZntR (55). Zur, a member of the Fur (ferric uptake regulator) family, typically binds with zinc to inhibit transcription (55). When zinc is deficient, Zur dissociates from zinc and upregulates ZnuABC expression to enhance zinc uptake (56). ZntR, which was initially identified in *E. coli,* is a MerR family transcriptional regulator (32). In conditions of zinc excess, ZntR binds excess zinc and activates ZntA expression to efflux zinc, maintaining zinc homeostasis (23, 34, 47–49). Zur and ZntR collaborate to regulate intracellular zinc homeostasis, preventing both zinc toxicity and deficiency.

Our previous study showed that intracellular zinc of *R. anatipestifer* CH-1 is the third most abundant transition metal (Fe^2+^ > Mn^2+^ > Zn^2+^ > Cu^2+^ > Ni^2+^ > Co^2+^) (43), highlighting its importance as a trace element. However, zinc homeostasis in *R. anatipestifer* has not been studied. This study confirmed that *B739_RS08595* (renamed as *zntR*) was involved in zinc homeostasis as a regulator. Transcriptome and proteome analyses revealed that *B739_RS07625* (renamed as *zupT*) is inhibited by ZntR and involved in zinc uptake.

In this study, we showed that Δ*zntR* exhibited significant growth defects in TSB compared to the WT (Fig. 1A). However, *zntR* deletion had no effect on the growth in *A. tumefaciens* and *B. abortus* (47, 48). The possible reasons for this difference include increased intracellular zinc content in Δ*zntR* when cultured in TSB (Fig. 2A), adversely affecting growth and the disruption of other metabolic pathways in *R. anatipestifer* (File S2). Zinc poisoning has been demonstrated to inhibit the synthesis of iron-sulfur clusters in *E. coli* (57). In this study, the *B739_RS03440* and *B739_RS03695* encoding iron-sulfur cluster assembly proteins were downregulated in Δ*zntR* (Table S4). The disruption of zinc homeostasis in *ΔzntR* affects these genes and warrants further investigation of their role in *R. anatipestifer*.

The type IX secretion system (T9SS) is a transmembrane multiprotein complex distributed in *Bacteroidetes* (58). T9SS has been shown to participate in the uptake of metals, such as Ca(II) and Mg(II), in *Cytophaga hutchinsonii* (59) but has not been reported to be involved in zinc uptake. In this study, we showed that T9SS type A sorting domain-containing proteins, B739_RS00425 and B739_RS00445, were downregulated in Δ*zntR* (Table S4), suggesting that T9SS has a potential zinc uptake function in *R. anatipestifer*. As an analogy, *Y. pseudotuberculosis* employs the T6SS effector YezP combined with the TonB-dependent receptor HumR to absorb zinc (60). In this study, the TonB-dependent receptors B739_RS02960, B739_RS06605, B739_RS00430, B739_RS00475, B739_RS05255, B739_RS01970, B739_RS00805, and B739_RS04305 were downregulated in Δ*zntR* (Table S4), suggesting these receptors are potentially involved in zinc homeostasis. The RND system has been shown to be involved in zinc and other metal efflux in *A. baumannii* as well as other bacteria (24, 61). Here, the RND efflux pump components B739_RS03800, B739_RS03805, B739_RS08825, B739_RS08830, and B739_RS08835 were upregulated in Δ*zntR* (Table S4). Actually, it has been shown that the RND efflux pump component *B739_RS04270* (*tolCA*) is involved in the efflux of six different metals (62), whereas *B739_RS08830* (*tolCB*) and *B739_RS08835* (*metB*) are specifically involved in manganese efflux (42, 62).

The ZIP (Zrt/Irt-like protein) family proteins are involved in zinc uptake and are widely found in bacteria, archaebacteria, and eukaryotes (63). In bacteria, the first member of this family was identified as a zinc uptake system in *E. coli* called ZupT (51). In this study, we observed that *B739_RS07625*, annotated as a ZIP family metal transporter, was upregulated in Δ*zntR* through transcriptome data and verified by qRT-PCR. It was then demonstrated that *B739_RS07625* is involved in zinc uptake by spot assay and ICP-MS, leading to its renaming as *zupT*. In *R. anatipestifer*, the growth of Δ*zupT* was impaired due to its reduced ability to uptake zinc (Fig. 5B and C). However, in *S. enterica* and *C. difficile*, only under severe zinc restriction conditions, the *zupT* deletion strain exhibits growth defects (15, 16). This difference suggests that ZupT is important for the growth of *R. anatipestifer*.

Zinc can protect sulfhydryl groups from oxidation and inhibit the production of ROS by transition metals (47). Moreover, zinc also serves as a cofactor of superoxide dismutase and induces the expression of metallothionein, enhancing the bacterial ability to eliminate reactive oxygen (64). The deletion of *zntR* resulted in increased tolerance to oxidative stress in *R. anatipestifer*. The possible reasons for this include (i) increased intracellular zinc content in Δ*zntR* in TSB enhances resistance to oxidative stress; (ii) upregulation of *zupT,* which is critical for oxidative stress resistance, in Δ*zntR* (Fig. 5E); (iii) upregulation of the catalase KatE (*B739_RS02180*) in Δ*zntR* (Table S4).

Host macrophages can mobilize cellular zinc to create an excess zinc environment for engulfed bacteria (65). Impaired zinc efflux capacity in bacteria hinders their growth within the host. To determine whether *zntR* deletion affects the virulence of *R. anatipestifer*, we performed lethality and colonization assays on ∆*zntR*. The results showed decreased colonization abilities of ∆*zntR* in ducklings. Taken together, our study confirms that ZntR of *R. anatipestifer* is involved in maintaining zinc homeostasis and contributes to virulence. This study is crucial for understanding zinc homeostasis mechanisms in zinc-limited and excessive environments.

## Data Availability

The transcriptomic data of *R. anatipestifer* CH-1 and CH-1 Δ*zntR* were uploaded to the National Center for Biotechnology Information Gene Expression Omnibus (NCBI GEO) database (accession number: GSE286539). The proteomic data of *R. anatipestifer* CH-1 and CH-1 Δ*zntR* were uploaded to the Proteomics Identifications database (accession number: PXD055543). The GenBank accession number of the whole genome of *R. anatipestifer* CH-1 is GCA_000295655.1. The GenBank accession numbers of ZntR for each species are as follows: *R. anatipestifer* (NCBI accession no. WP_004918800.1), *E. coli* (NCBI accession no. NP_417751.1), *Y*. *pseudotuberculosis* (NCBI accession no. WP_002215702.1), *V. parahaemolyticus* (NCBI accession no. WP_005456436.1), *S*. *aureus* (NCBI accession no. WP_000850000.1), *A. tumefaciens* (NCBI accession no. WP_010971227.1), *C. metallidurans* (NCBI accession no. WP_008646161.1), and *B. abortus* (NCBI accession no. WP_002965082.1). The GenBank accession numbers of ZupT for each species are as follows: *R. anatipestifer* (NCBI accession no. WP_004920108.1), *C. difficile* (NCBI accession no. WP_009892895.1), *S. enterica* (NCBI accession no. NP_462105.1), *E. coli* (NCBI accession no. NP_417512.1), and *C. metallidurans* (NCBI accession no. WP_186424897.1).

## SUPPLEMENTAL MATERIAL

Fig. S1 spectrum.03178-24-s0001.tif Alignment of ZntR amino acid sequences from different species.

Fig. S2 spectrum.03178-24-s0002.tif Alignment of ZupT amino acid sequences from different species.

Fig. S3 spectrum.03178-24-s0003.tif The deletion of *zntR* did not lead to the accumulation of Mn, Cu, Fe, Co and Ni in cells.

Supplemental material spectrum.03178-24-s0004.docx Tables S1 to S5; Supplemental figure captions.

Supplemental data spectrum.03178-24-s0005.xlsx Transcriptome and proteome data.

ASM does not own the copyrights to Supplemental Material that may be linked to, or accessed through, an article. The authors have granted ASM a non-exclusive, world-wide license to publish the Supplemental Material files. Please contact the corresponding author directly for reuse.
