# Supplementary figures and images for: ZntR is a critical regulator for zinc homeostasis and involved in pathogenicity in Riemerella anatipestifer
Source: Microbiol Spectr. 2025 Mar 4;13(4):e03178-24. doi: 10.1128/spectrum.03178-24 (PMC11960050; doi:10.1128/spectrum.03178-24)

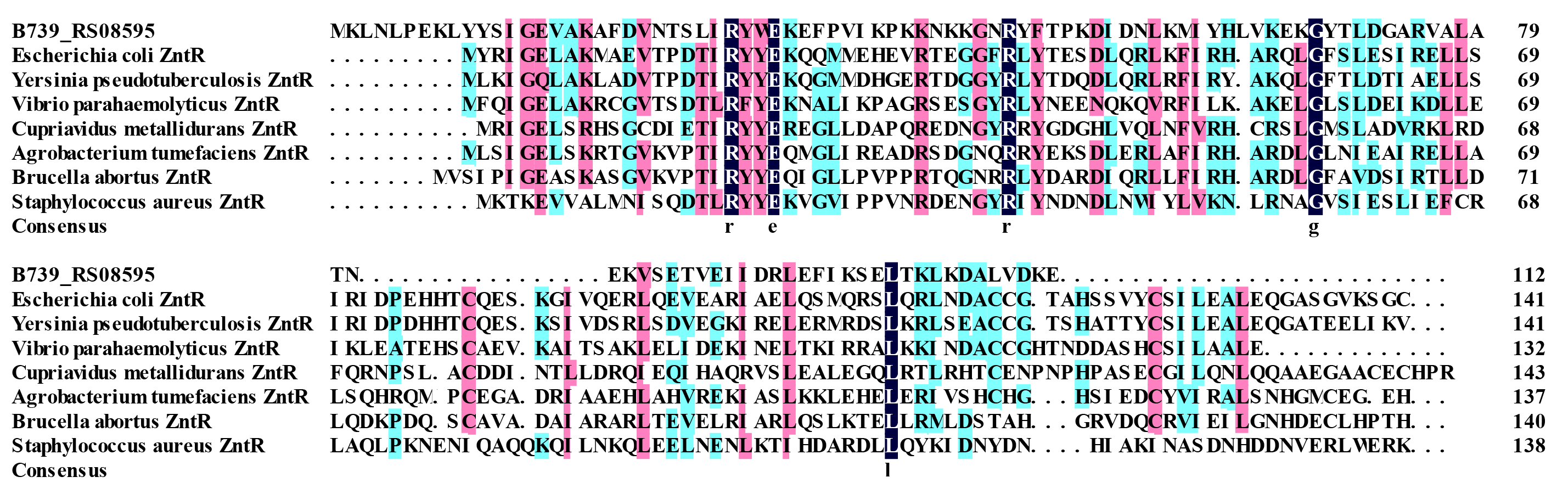

Supplement: Fig. S1 — Alignment of ZntR amino acid sequences from different species. [file spectrum.03178-24-s0001.tif]

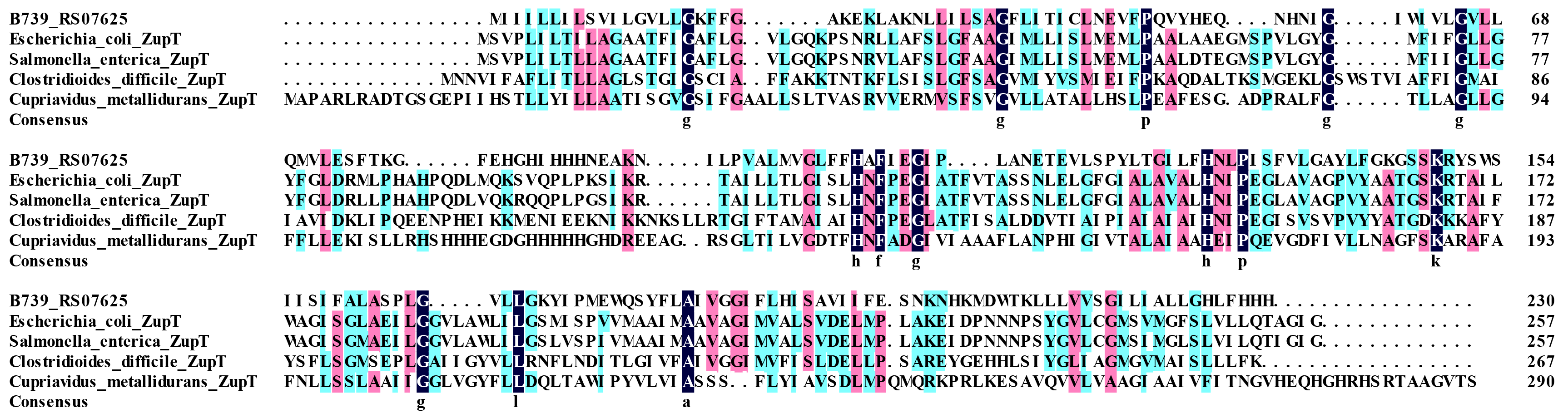

Supplement: Fig. S2 — Alignment of ZupT amino acid sequences from different species. [file spectrum.03178-24-s0002.tif]

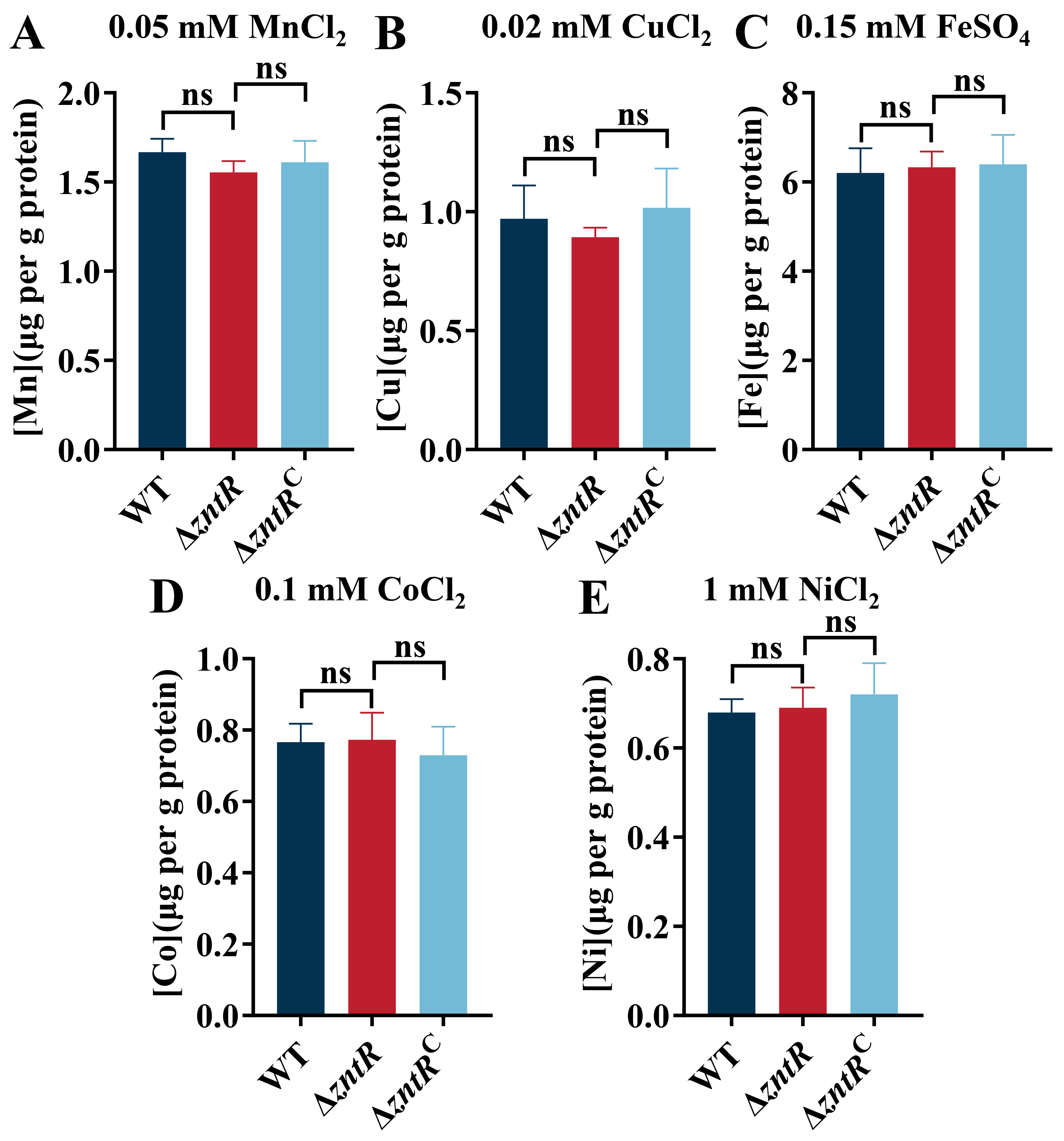

Supplement: Fig. S3 — The deletion of zntR did not lead to the accumulation of Mn, Cu, Fe, Co and Ni in cells. [file spectrum.03178-24-s0003.tif]
