# Supplementary material for: ZntR is a critical regulator for zinc homeostasis and involved in pathogenicity in Riemerella anatipestifer
Source: Microbiol Spectr. 2025 Mar 4;13(4):e03178-24. doi: 10.1128/spectrum.03178-24 (PMC11960050; doi:10.1128/spectrum.03178-24)
Supplement: Supplemental material — Tables S1 to S5; Supplemental figure captions. [file spectrum.03178-24-s0004.docx]

Supplemental Material

**TABLE S1. Strains and plasmids used in this study**

| **Strains** | **Phenotype or genotype** | **Source or reference** |
| --- | --- | --- |
| *E. coli* DH5α | F^-^*,φ80dlacZ*Δ*M15,*Δ(*lacZYA-argF*)*U169, deoR, recA1, endA1, hsdR17 (rK^-^, mK^+^), phoA, supE44, λ^-^, thi-1, gyrA96*, *relA1* | Laboratory collection |
| *E. coli* S17-1 | *hsdR17 recA1* RP4-2-tet::Mu-1kan::Tn7; Sm^R^ | Laboratory collection |
| *R. anatipestifer* CH-1 | *R. anatipestifer serotype* 1, Km^R^, Erm^R^ | Laboratory collection |
| *R. anatipestifer* CH-1 pLMF02 | *R. anatipestifer serotype* 1, pLMF02*,* Cfx^R^ | This study |
| *R. anatipestifer* CH-1Δ*zntR::spc* pLMF02 | *R. anatipestifer* CH-1, *zntR* mutant, Spc^R^, pLMF02*,* Cfx^R^ | This study |
| *R. anatipestifer* CH-1Δ*zntR::spc* pLMF02*::zntR* | *R. anatipestifer* CH-1, *zntR* mutant, Spc^R^, pLMF02*::zntR,* Cfx^R^ | This study |
| *R. anatipestifer* CH-1Δ*zupT* pLMF02 | *R. anatipestifer* CH-1, *zupT* mutant, pLMF02*,* Cfx^R^ | This study |
| *R. anatipestifer* CH-1Δ*zupT* pLMF02*::zupT* | *R. anatipestifer* CH-1, *zupT* mutant, Spc^R^, pLMF02*::zupT,* Cfx^R^ | This study |
| **Plasmids** | **Phenotype or Genotype** | **Source or reference** |
| pLMF02 | Shuttle plasmid, *ompA* promoter, *ori*ColE1, *ori*pRA0726, Amp^R^, Cfx^R^ | Laboratory collection |
| pBAD24::*cfx-sacB* | pBAD24 carrying *cfx* from pLMF02 and *sacB* from pEX18GM, Amp^R^, Cfx^R^ | Laboratory collection |
| pBAD24::*zupT* up-*cfx-sacB-zupT down* | pBAD24 carrying *zupT* upstream and *zupT* downstream, carrying *cfx* from pLMF02 and *sacB* from pEX18GM, Amp^R^, Cfx^R^ | This study |
| pLMF02*::zntR* | pLMF02 carrying *zntR* from *R. anatipestifer* CH-1, Amp^R^, Cfx^R^ | This study |
| pLMF02*::zupT* | pLMF02 carrying *zupT* from *R. anatipestifer* CH-1, Amp^R^, Cfx^R^ | This study |

Km^R^, kanamycin resistance; Amp^R^, ampicillin resistance; Erm^R^, erythromycin resistance; Cfx^R^, cefoxitin resistance; Spc^R^, spectinomycin resistance.

**TABLE S2. Primers used in this study**

| **Primers** | **Sequence (5′-3′)** | **Organism** |
| --- | --- | --- |
| *zntR* up P1 | GTACGAAGTGGTTAATAAATTTCATAAAATGCTAGAAACC | *R. anatipestifer* CH-1 |
| *zntR* up P2 | TTATAACATGTATTCACGAACAACTTCGCCTATAGAATAA | *R. anatipestifer* CH-1 |
| *zntR* spc P1 | TTATTCTATAGGCGAAGTTGTTCGTGAATACATGTTATAA | pAM238 |
| *zntR* spc P2 | CTCTAGTCGGTCTATTATTTGCTTACCAATTAGAATGAAT | pAM238 |
| *zntR* down P1 | ATTCATTCTAATTGGTAAGCAAATAATAGACCGACTAGAG | *R. anatipestifer* CH-1 |
| *zntR* down P2 | ACTTGCTTTTTTATCATACTGAACTCCAGTTTAGAGAATG | *R. anatipestifer* CH-1 |
| *zntR* comp P1 | CATGCCATGGATGAAACTTAATCTTCCAG | *R. anatipestifer* CH-1 |
| *zntR* comp P2 | CCGCTCGAGTTACTCCTTATCTACCAAAG | *R. anatipestifer* CH-1 |
| *zupT* up P1 | CGGAATTCCAATAATTTTGATATTAGGG | *R. anatipestifer* CH-1 |
| *zupT* up P2 | GGGGTACCAGAATTAAAAATAGAACCGC | *R. anatipestifer* CH-1 |
| *zupT* down P1 | ACGCGTCGACCATTAAAGACTAAATCTGAT | *R. anatipestifer* CH-1 |
| *zupT* down P2 | AACTGCAGGGCAGATTTGGAAGCACAAC | *R. anatipestifer* CH-1 |
| *zupT* F up P1 | ATTTTAGAAGGTATAGAAAAGTCTTCTTTAAAAATAGGAG | *R. anatipestifer* CH-1 |
| *zupT* F up P2 | CAATCAGATTTAGTCTTTAAATAGAATTAAAAATAGAACC | *R. anatipestifer* CH-1 |
| *zupT* F down P1 | GGTTCTATTTTTAATTCTATTTAAAGACTAAATCTGATTG | *R. anatipestifer* CH-1 |
| *zupT* F down P2 | GAGATTGTGTATTTCGTTAATCAAACAAGTAATAATAG | *R. anatipestifer* CH-1 |
| *zupT* comp P1 | ACGCGTCGACATAAGACTTAAATATCTG | *R. anatipestifer* CH-1 |
| *zupT* comp P2 | CCGCTCGAGTTAATGATGATGGAATAAATG | *R. anatipestifer* CH-1 |
| *zupT* q P1 | TCCCTTTAGCTAACGAAACAG | *R. anatipestifer* CH-1 |
| *zupT* q P2 | CCTTTACCAAACAAATACGCTC | *R. anatipestifer* CH-1 |
| 16S rRNA q P1 | ATGCGAAAGGAGGATTGC | *R. anatipestifer* CH-1 |
| 16S rRNA q P2 | TTACACCTCAAATACCTC | *R. anatipestifer* CH-1 |
| *cfx* F | TTTCATTGTTCCATAAATCAGC | pLMF02 |
| *cfx* R | TACACTGTTTTGCATTCTTTT | pLMF02 |
| 16S rRNA P1 | CTTCGGATACTTGAGAGCG | *R. anatipestifer* CH-1 |
| 16S rRNA P2 | GCAGCACCTTGAAAATTGT | *R. anatipestifer* CH-1 |

The underline indicates restriction enzyme sites

**TABLE S3. DEGs that are potentially related to zinc metabolism**

| **seq_id** | **fc** | **log_2_fc** | **significant** | **regulate** | **description** |
| --- | --- | --- | --- | --- | --- |
| B739_RS00805 | 19.78955 | 4.306667 | yes | up | TonB-dependent receptor |
| B739_RS00475 | 8.779857 | 3.134197 | yes | up | SusC/RagA family TonB-linked outer membrane protein |
| B739_RS05255 | 8.144653 | 3.025853 | yes | up | TonB-dependent receptor |
| B739_RS00430 | 4.08885 | 2.031695 | yes | up | TonB-dependent receptor |
| B739_RS01550 | 4.056353 | 2.020183 | yes | up | TonB-dependent receptor |
| B739_RS01890 | 3.496702 | 1.805995 | yes | up | TonB-dependent receptor |
| B739_RS06990 | 3.144276 | 1.652728 | yes | up | TonB-dependent receptor |
| B739_RS00320 | 2.646627 | 1.404155 | yes | up | TonB-dependent receptor |
| B739_RS04295 | 2.042113 | 1.030063 | yes | up | SusC/RagA family TonB-linked outer membrane protein |
| B739_RS00540 | 2.009577 | 1.006892 | yes | up | SusC/RagA family TonB-linked outer membrane protein |
| B739_RS00425 | 8.516582 | 3.090274 | yes | up | T9SS type A sorting domain-containing protein |
| B739_RS06145 | 5.058392 | 2.338679 | yes | up | T9SS type A sorting domain-containing protein |
| B739_RS00445 | 4.59898 | 2.201314 | yes | up | T9SS type A sorting domain-containing protein |
| B739_RS07625 | 2.307832 | 1.206538 | yes | up | ZIP family metal transporter |
| B739_RS00465 | 6.988407 | 2.804964 | yes | up | putative zinc-binding metallopeptidase |
| B739_RS09255 | 2.60782 | 1.382844 | yes | up | fibronectin type III domain-containing protein |
| B739_RS08645 | 2.60437 | 1.380934 | yes | up | M14 family zinc carboxypeptidase |
| B739_RS02910 | 6.197392 | 2.631661 | yes | up | ferrous iron transport protein A |
| B739_RS03695 | 6.215094 | 2.635776 | yes | up | iron-sulfur cluster assembly accessory protein |
| B739_RS02540 | 0.188531 | -2.40713 | yes | down | TonB-dependent receptor |
| B739_RS03865 | 0.262657 | -1.92875 | yes | down | SusC/RagA family TonB-linked outer membrane protein |
| B739_RS05435 | 0.298725 | -1.74311 | yes | down | SusC/RagA family TonB-linked outer membrane protein |
| B739_RS02835 | 0.402225 | -1.31393 | yes | down | SusC/RagA family TonB-linked outer membrane protein |
| B739_RS02960 | 0.481996 | -1.05291 | yes | down | TonB-dependent receptor |
| B739_RS01970 | 0.408168 | -1.29277 | yes | down | TonB-dependent receptor |
| B739_RS08835 | 0.299424 | -1.73974 | yes | down | CusA/CzcA family heavy metal efflux RND transporter |
| B739_RS08830 | 0.375845 | -1.41179 | yes | down | TolC family protein |
| B739_RS08825 | 0.412587 | -1.27723 | yes | down | efflux RND transporter periplasmic adaptor subunit |
| B739_RS04275 | 0.450604 | -1.15007 | yes | down | efflux RND transporter periplasmic adaptor subunit |
| B739_RS09300 | 0.368356 | -1.44083 | yes | down | insulinase family protein |
| B739_RS10930 | 0.368752 | -1.43928 | yes | down | zinc ribbon domain-containing protein |
| B739_RS02180 | 0.408324 | -1.29221 | yes | down | catalase |

**TABLE S4. DAPs that are potentially related to zinc metabolism**

| **seq_id** | **fc** | **log_2_fc** | **P value** | **regulate** | **description** |
| --- | --- | --- | --- | --- | --- |
| B739_RS01470 | 1.692 | 0.7587 | 0.001304 | up | SusC/RagA family TonB-linked outer membrane protein |
| B739_RS02540 | 1.633 | 0.7075 | 0.0002478 | up | Outer membrane receptor protein, mostly Fe transport |
| B739_RS10310 | 1.63 | 0.7049 | 0.001581 | up | Outer membrane receptor protein, mostly Fe transport |
| B739_RS04255 | 1.498 | 0.583 | 0.003235 | up | TonB C-terminal domain-containing protein |
| B739_RS05365 | 1.429 | 0.515 | 0.003958 | up | TonB-dependent receptor-like beta-barrel domain-containing protein |
| B739_RS01235 | 1.404 | 0.4895 | 0.001203 | up | Outer membrane receptor protein, mostly Fe transport |
| B739_RS10585 | 1.33 | 0.4114 | 0.0009872 | up | TonB-dependent receptor plug domain-containing protein |
| B739_RS08825 | 2.165 | 1.114 | 0.0008402 | up | RND efflux pump membrane fusion protein barrel-sandwich domain-containing protein |
| B739_RS08835 | 2.157 | 1.109 | 0.002719 | up | Putative silver efflux pump |
| B739_RS08830 | 1.783 | 0.8343 | 0.004855 | up | Outer membrane protein |
| B739_RS10780 | 1.568 | 0.6489 | 0.006287 | up | Na+-driven multidrug efflux pump |
| B739_RS03800 | 1.49 | 0.5753 | 0.001043 | up | Cation/multidrug efflux pump |
| B739_RS03805 | 1.429 | 0.515 | 0.003402 | up | RND efflux pump membrane fusion protein barrel-sandwich domain-containing protein |
| B739_RS01815 | 1.356 | 0.4394 | 0.003683 | up | Cation transport ATPase |
| B739_RS08645 | 1.897 | 0.9237 | 8.10E-05 | up | Peptidase M14 carboxypeptidase A domain-containing protein |
| B739_RS00590 | 1.751 | 0.8082 | 0.006321 | up | HIRAN domain-containing protein |
| B739_RS02180 | 1.521 | 0.605 | 0.007902 | up | Catalase |
| B739_RS01475 | 1.415 | 0.5008 | 0.002912 | up | Metalloprotease |
| B739_RS02960 | 0.7255 | -0.463 | 0.0002403 | down | HMA domain-containing protein |
| B739_RS06605 | 0.7239 | -0.4661 | 0.02509 | down | Outer membrane receptor protein, mostly Fe transport |
| B739_RS00430 | 0.6275 | -0.6723 | 0.0305 | down | Outer membrane receptor for Fe3+-dicitrate |
| B739_RS00475 | 0.5456 | -0.8741 | 0.03127 | down | TonB-dependent receptor plug domain-containing protein |
| B739_RS05255 | 0.5185 | -0.9476 | 0.03662 | down | Outer membrane receptor protein, mostly Fe transport |
| B739_RS00805 | 0.5154 | -0.9562 | 0.01623 | down | Outer membrane receptor protein, mostly Fe transport |
| B739_RS01970 | 0.6857 | -0.5444 | 0.003041 | down | outer membrane beta-barrel protein |
| B739_RS04305 | 0.1472 | -2.764 | 0.01599 | down | TonB-dependent receptor plug domain-containing protein |
| B739_RS09300 | 0.7697 | -0.3776 | 0.0004833 | down | Zn-dependent peptidase |
| B739_RS05660 | 0.7258 | -0.4624 | 0.04265 | down | Putative Zn-dependent protease |
| B739_RS03715 | 0.6972 | -0.5204 | 0.001287 | down | DnaK suppressor protein |
| B739_RS00465 | 0.4682 | -1.095 | 0.01085 | down | DUF1570 domain-containing protein |
| B739_RS00425 | 0.2904 | -1.784 | 0.02209 | down | Secretion system C-terminal sorting domain-containing protein |
| B739_RS03695 | 0.6711 | -0.5754 | 0.03817 | down | FeS cluster biogenesis domain-containing protein |
| B739_RS03440 | 0.7444 | -0.4259 | 0.005415 | down | SufE protein probably involved in Fe-S center assembly |
| B739_RS02910 | 0.3371 | -1.569 | 0.006614 | down | Ferrous iron transporter FeoA domain-containing protein |
| B739_RS00445 | 0.3863 | -1.372 | 0.03632 | down | CBM-cenC domain-containing protein |

**TABLE S5. Genes and proteins that are upregulated or downregulated in both transcriptomic and proteomic analyses.**

| **seq_id** | **transcriptome** | | **proteome** | | **description** |
| --- | --- | --- | --- | --- | --- |
|  | **fc** | **log_2_fc** | **fc** | **log_2_fc** |  |
| B739_RS06690 | 26.1828 | 4.710547 | 13.61 | 3.767 | hypothetical protein |
| B739_RS02350 | 18.94744 | 4.243931 | 22.58 | 4.497 | DUF6175 family protein |
| B739_RS02355 | 18.16683 | 4.183235 | 28.9 | 4.853 | hypothetical protein |
| B739_RS02360 | 17.20774 | 4.104986 | 22.36 | 4.483 | hypothetical protein |
| B739_RS06695 | 15.63164 | 3.966397 | 63.66 | 5.992 | hypothetical protein |
| B739_RS02345 | 14.15301 | 3.823037 | 27.78 | 4.796 | hypothetical protein |
| B739_RS00590 | 13.89691 | 3.796692 | 1.751 | 0.8082 | HIRAN domain-containing protein |
| B739_RS06685 | 10.55955 | 3.400477 | 14.22 | 3.83 | hypothetical protein |
| B739_RS02365 | 9.920449 | 3.310405 | 31.83 | 4.992 | hypothetical protein |
| B739_RS00595 | 6.869943 | 2.780298 | 1.336 | 0.4179 | RtcB family protein |
| B739_RS09940 | 5.894759 | 2.559433 | 2.038 | 1.027 | CPBP family intramembrane metalloprotease |
| B739_RS05775 | 3.978584 | 1.992255 | 1.662 | 0.7329 | hypothetical protein |
| B739_RS02000 | 3.571701 | 1.836611 | 1.761 | 0.8164 | hypothetical protein |
| B739_RS05785 | 3.465431 | 1.793035 | 1.633 | 0.7075 | transporter |
| B739_RS08785 | 3.342466 | 1.740913 | 1.399 | 0.4844 | hypothetical protein |
| B739_RS05780 | 3.342177 | 1.740788 | 1.615 | 0.6915 | c-type cytochrome |
| B739_RS07395 | 2.997505 | 1.583762 | 1.816 | 0.8608 | DUF4469 domain-containing protein |
| B739_RS08645 | 2.60437 | 1.380934 | 1.897 | 0.9237 | M14 family zinc carboxypeptidase |
| B739_RS08730 | 2.469668 | 1.304317 | 1.3 | 0.3785 | YtxH domain-containing protein |
| B739_RS00315 | 2.393361 | 1.259038 | 1.332 | 0.4136 | sensor histidine kinase |
| B739_RS05875 | 2.271544 | 1.183673 | 1.459 | 0.545 | DUF4199 domain-containing protein |
| B739_RS00835 | 2.201785 | 1.138674 | 1.372 | 0.4563 | YigZ family protein |
| B739_RS08595 | 0.021797 | -5.5197 | 0 | -16.61 | MerR family transcriptional regulator |
| B739_RS05430 | 0.2494 | -2.00347 | 0.7309 | -0.4523 | ribonuclease HII |
| B739_RS07745 | 0.331974 | -1.59086 | 0.6649 | -0.5888 | aspartate carbamoyltransferase catalytic subunit |
| B739_RS09300 | 0.368356 | -1.44083 | 0.7697 | -0.3776 | insulinase family protein |
| B739_RS04410 | 0.376689 | -1.40856 | 0.7506 | -0.4139 | GNAT family N-acetyltransferase |
| B739_RS01595 | 0.402424 | -1.31321 | 0.4963 | -1.011 | hypothetical protein |
| B739_RS01970 | 0.408168 | -1.29277 | 0.6857 | -0.5444 | outer membrane beta-barrel protein |
| B739_RS07025 | 0.435396 | -1.1996 | 0.7607 | -0.3946 | universal stress protein |
| B739_RS05310 | 0.440126 | -1.18401 | 0.7049 | -0.5045 | DUF2480 family protein |
| B739_RS06445 | 0.459583 | -1.1216 | 0.3822 | -1.388 | hypothetical protein |
| B739_RS03295 | 0.462613 | -1.11212 | 0.7208 | -0.4723 | 6,7-dimethyl-8-ribityllumazine synthase |
| B739_RS02960 | 0.481996 | -1.05291 | 0.7255 | -0.463 | TonB-dependent receptor |

**Fig. S1 Alignment of ZntR amino acid sequences from different species.**

**
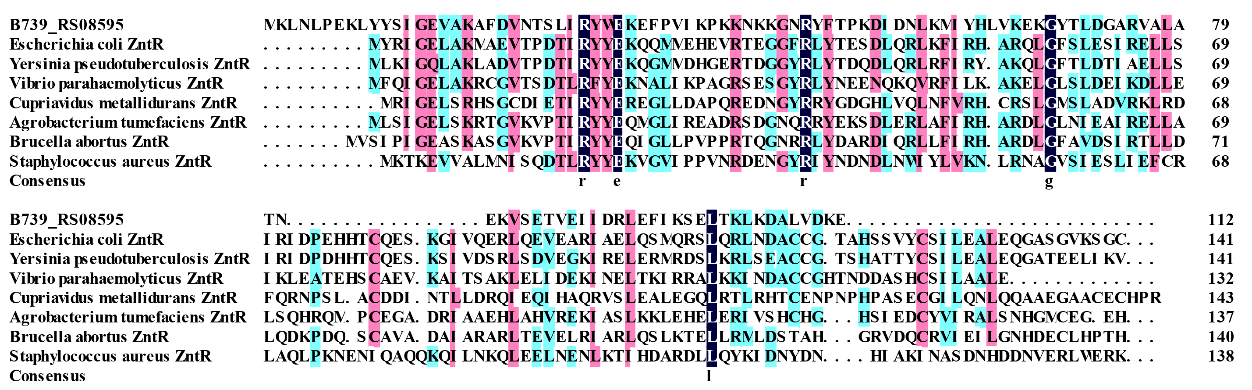
**

**Fig. S1 Alignment of ZntR amino acid sequences from different species.** The *B739_RS08595* shares 10.79-21.19% identity with ZntR proteins from *Escherichia coli*, *Vibrio parahaemolyticus*, *Yersinia pseudotuberculosis*, *Brucella abortus*, *Agrobacterium tumefaciens*, *Cupriavidus metallidurans* and *Staphylococcus aureus* respectively. Multiple sequence alignment colored by amino acid homology. Darker colors indicate higher homology. Black represents 100% homology, pink represents ≥75%, and cyan represents ≥50% homology. The identities were as follows: 21.19% with *Escherichia coli*(1), 21.13% with *Vibrio parahaemolyticus*(2), 19.87% with *Yersinia pseudotuberculosis*(3), 19.59% with *Brucella abortus*(4), 17.69% with *Agrobacterium tumefaciens*(5), 13.64% with *Cupriavidus metallidurans*(6), 10.79% with *Staphylococcus aureus*(7). The alignment was performed using DNAMAN software.

**Fig. S2 Alignment of ZupT amino acid sequences from different species.**


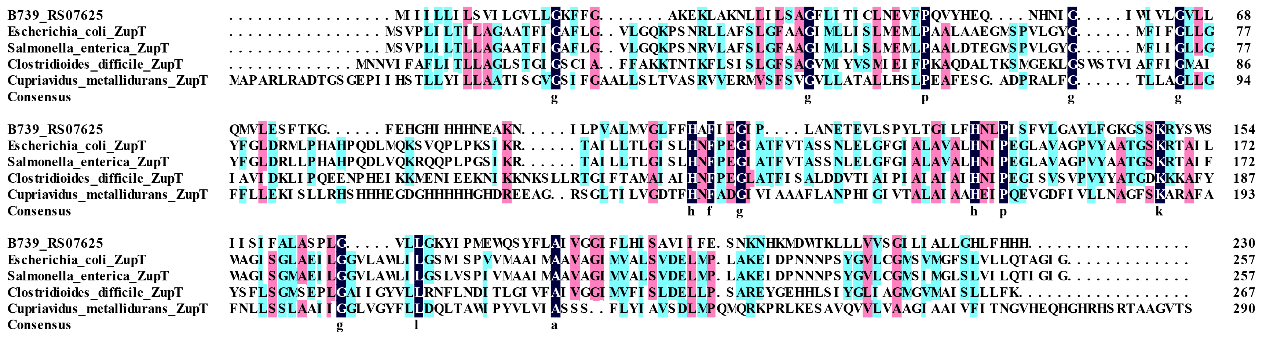


**Fig. S2 Alignment of ZupT amino acid sequences from different species.** The *B739_RS07625* shares 17.87-19.77% identity with ZupT proteins from *Escherichia coli*, *Salmonella enterica*, *Clostridioides difficile* and *Cupriavidus metallidurans* respectively. Multiple sequence alignment colored by amino acid homology. Darker colors indicate higher homology. Black represents 100% homology, pink represents ≥75%, and cyan represents ≥50% homology. The identities were as follows: 19.77% with *Escherichia coli*(8), 19.38% with *Salmonella enterica*(9), 17.91% with *Clostridioides difficile*(10), 17.87% with *Cupriavidus metallidurans*(11). The alignment was performed using DNAMAN software.

**Fig. S3: The deletion of *zntR* did not lead to the accumulation of Mn, Cu, Fe, Co and Ni in cells.**


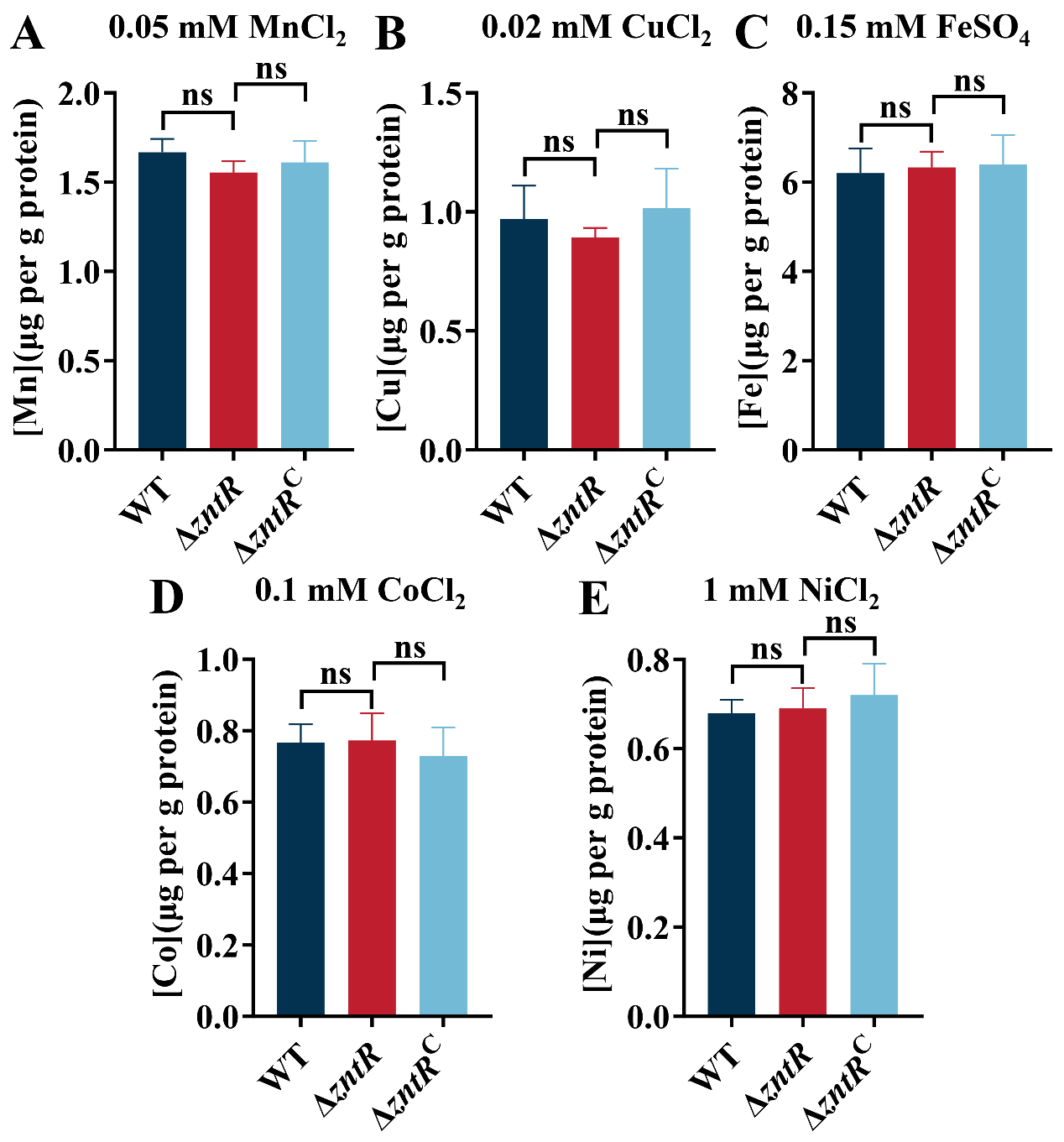


**Fig. S3: The deletion of *zntR* did not lead to the accumulation of Mn, Cu, Fe, Co and Ni in cells. A.** Intracellular Mn content of WT, Δ*zntR*, and Δ*zntR^C^* in TSB supplemented by 0.05 mM MnCl_2_. **B.** Intracellular Cu content of WT, Δ*zntR*, and Δ*zntR^C^* in TSB supplemented by 0.02 mM CuCl_2_. **C.** Intracellular Fe content of WT, Δ*zntR*, and Δ*zntR^C^* in TSB supplemented by 0.15 mM FeSO_4_. **D.** Intracellular Co content of WT, Δ*zntR*, and Δ*zntR^C^* in TSB supplemented by 0.1 mM CoCl_2_. **E.** Intracellular Ni content of WT, Δ*zntR*, and Δ*zntR^C^* in TSB supplemented by 1 mM NiCl_2_. Data represent means and standard deviations of results from 3 independent experiments. Significant differences compared to the WT were determined by one-way ANOVA. The P values of the data were calculated by one-way ANOVA using GraphPad Prism 8. Asterisks denote significant differences n.s., no significant difference.

**References**

1. Brocklehurst KR, Hobman JL, Lawley B, Blank L, Marshall SJ, Brown NL, et al. ZntR is a Zn(II)-responsive MerR-like transcriptional regulator of zntA in Escherichia coli. Molecular microbiology. 1999;31(3):893-902. Epub 1999/02/27. doi: 10.1046/j.1365-2958.1999.01229.x. PubMed PMID: 10048032.

2. Zheng C, Zhai Y, Qiu J, Wang M, Xu Z, Chen X, et al. ZntA maintains zinc and cadmium homeostasis and promotes oxidative stress resistance and virulence in Vibrio parahaemolyticus. Gut microbes. 2024;16(1):2327377. Epub 2024/03/11. doi: 10.1080/19490976.2024.2327377. PubMed PMID: 38466137; PubMed Central PMCID: PMC10936601.

3. Wang T, Chen K, Gao F, Kang Y, Chaudhry MT, Wang Z, et al. ZntR positively regulates T6SS4 expression in Yersinia pseudotuberculosis. Journal of microbiology (Seoul, Korea). 2017;55(6):448-56. Epub 2017/03/11. doi: 10.1007/s12275-017-6540-2. PubMed PMID: 28281200.

4. Sheehan LM, Budnick JA, Roop RM, 2nd, Caswell CC. Coordinated zinc homeostasis is essential for the wild-type virulence of Brucella abortus. Journal of bacteriology. 2015;197(9):1582-91. Epub 2015/02/19. doi: 10.1128/jb.02543-14. PubMed PMID: 25691532; PubMed Central PMCID: PMC4403653.

5. Chaoprasid P, Nookabkaew S, Sukchawalit R, Mongkolsuk S. Roles of Agrobacterium tumefaciens C58 ZntA and ZntB and the transcriptional regulator ZntR in controlling Cd2+/Zn2+/Co2+ resistance and the peroxide stress response. Microbiology (Reading, England). 2015;161(9):1730-40. Epub 2015/08/25. doi: 10.1099/mic.0.000135. PubMed PMID: 26296876.

6. Schulz V, Schmidt-Vogler C, Strohmeyer P, Weber S, Kleemann D, Nies DH, et al. Behind the shield of Czc: ZntR controls expression of the gene for the zinc-exporting P-type ATPase ZntA in Cupriavidus metallidurans. Journal of bacteriology. 2021;203(11). Epub 2021/03/10. doi: 10.1128/jb.00052-21. PubMed PMID: 33685972; PubMed Central PMCID: PMC8117531.

7. Singh VK, Xiong A, Usgaard TR, Chakrabarti S, Deora R, Misra TK, et al. ZntR is an autoregulatory protein and negatively regulates the chromosomal zinc resistance operon znt of Staphylococcus aureus. Molecular microbiology. 1999;33(1):200-7. Epub 1999/07/20. doi: 10.1046/j.1365-2958.1999.01466.x. PubMed PMID: 10411736.

8. Grass G, Wong MD, Rosen BP, Smith RL, Rensing C. ZupT is a Zn(II) uptake system in Escherichia coli. Journal of bacteriology. 2002;184(3):864-6. Epub 2002/01/16. doi: 10.1128/jb.184.3.864-866.2002. PubMed PMID: 11790762; PubMed Central PMCID: PMC139533.

9. Cerasi M, Liu JZ, Ammendola S, Poe AJ, Petrarca P, Pesciaroli M, et al. The ZupT transporter plays an important role in zinc homeostasis and contributes to Salmonella enterica virulence. Metallomics : integrated biometal science. 2014;6(4):845-53. Epub 2014/01/17. doi: 10.1039/c3mt00352c. PubMed PMID: 24430377; PubMed Central PMCID: PMC3969385.

10. Zackular JP, Knippel RJ, Lopez CA, Beavers WN, Maxwell CN, Chazin WJ, et al. ZupT Facilitates Clostridioides difficile Resistance to Host-Mediated Nutritional Immunity. mSphere. 2020;5(2). Epub 2020/03/13. doi: 10.1128/mSphere.00061-20. PubMed PMID: 32161145; PubMed Central PMCID: PMC7067591.

11. Herzberg M, Bauer L, Nies DH. Deletion of the zupT gene for a zinc importer influences zinc pools in Cupriavidus metallidurans CH34. Metallomics : integrated biometal science. 2014;6(3):421-36. Epub 2014/01/11. doi: 10.1039/c3mt00267e. PubMed PMID: 24407051.
